# Population Genetic Studies Revealed Local Adaptation in a High Gene-Flow Marine Fish, the Small Yellow Croaker (*Larimichthys polyactis*)

**DOI:** 10.1371/journal.pone.0083493

**Published:** 2013-12-12

**Authors:** Le Wang, Shufang Liu, Zhimeng Zhuang, Liang Guo, Zining Meng, Haoran Lin

**Affiliations:** 1 State Key Laboratory of Biocontrol, Institute of Aquatic Economic Animals and the Guangdong Province Key Laboratory for Aquatic Economic Animals, School of Life Sciences, Sun Yat-sen University, Guangzhou, Guangdong, China; 2 Yellow Sea Fisheries Research Institute, Chinese Academy of Fishery Sciences, Qingdao, Shandong, China; Kunming Institute of Zoology, Chinese Academy of Sciences, China

## Abstract

The genetic differentiation of many marine fish species is low. Yet local adaptation may be common in marine fish species as the vast and changing marine environment provides more chances for natural selection. Here, we used anonymous as well as known protein gene linked microsatellites and mitochondrial DNA to detect the population structure of the small yellow croaker (*Larimichthys polyactis*) in the Northwest Pacific marginal seas. Among these loci, we detected at least two microsatellites, anonymous H16 and HSP27 to be clearly under diversifying selection in outlier tests. Sequence cloning and analysis revealed that H16 was located in the intron of BAHCC1 gene. Landscape genetic analysis showed that H16 mutations were significantly associated with temperature, which further supported the diversifying selection at this locus. These marker types presented different patterns of population structure: (i) mitochondrial DNA phylogeny showed no evidence of genetic divergence and demonstrated only one glacial linage; (ii) population differentiation using putatively neutral microsatellites presented a pattern of high gene flow in the *L. polyactis*. In addition, several genetic barriers were identified; (iii) the population differentiation pattern revealed by loci under diversifying selection was rather different from that revealed by putatively neutral loci. The results above suggest local adaptation in the small yellow croaker. In summary, population genetic studies based on different marker types disentangle the effects of demographic history, migration, genetic drift and local adaptation on population structure and also provide valuable new insights for the design of management strategies in *L. polyactis*.

## Introduction

In many marine fish species, especially migratory species, the signal of population differentiation is weak and hard to detect due to high levels of gene flow. It is traditionally difficult to delineate the population structure or detect the population subdivision lacking clear barriers to gene flow within ocean basins [1]. Especially in fishes of recent speciation, this problem has become more complicated because of little accumulation of genetic differentiation in such a short period of time [2]. However, advancement in population genetic studies has greatly improved our methods in elucidating the population genetic structure of marine fishes of high mobility and high dispersal potential during egg and larval stages [3]. Fisheries studies, electronic and microchemical tagging techniques provide direct information in analyzing population dynamics of some marine fishes. Nevertheless, the genetic approaches can suggest unique strategies for stock management and evolutionary unit conservation [4,5], proving to be particularly important for the economically important marine fish species facing high fishing pressure or already being threatened.

In recent years, a growing number of studies have revealed low but statistically significant differentiation among populations of many marine fishes using microsatellites, a highly variable and presumably neutral genetic marker [6-11]. Mitochondrial DNA (mtDNA), another marker system, was also employed to detect population divergence caused by historical isolation in some marine fishes in many other studies [11-13]. Even in some marine fishes with low or no differentiation, marked genetic structuring was also observed using directional selected markers [14]. However, due to different modes of inheritance and evolution, results based on different marker types may provide different insights for fishery management and conservation. Hypervariable microsatellites are considered more powerful in revealing recent evolutionary processes, but not suitable for phylogeographic study [15]. Conversely, mtDNA of slow mutation rates is more sensitive for detecting phylogeographical structure and historical evolutionary process due to its nonrecombinant and sex-biased inheritance [16]. However, population differentiation revealed by directional selected markers reflect the local adaptation to different environments, although the evolutionary mechanism of this marker is rather complicated and far from being understood. It was suggested that this differentiation could result from the effect of natural selection over the effect of random drift combined with the homogenizing effect of gene flow [17]. Therefore, studies of adaptive genetic divergence at spatial and temporal scale in marine species are crucial to improve our understanding of the evolutionary process [17-19]. These three marker types have different evolutionary mechanisms and can produce differing results, thus should be treated with caution when interpreting the results of population genetic analysis. Meanwhile, these approaches combined can obtain more accurate and comprehensive understanding of the features, dynamics and evolutionary processes of the marine fish species. 

Several previous studies have reported local selection in some marine fishes. For example, the haemoglobin locus showed differentiation along latitudinal cline in Atlantic cod (*Gadus morhua*) between the North Sea and the Baltic Sea [20,21]. European ﬂounder (*Platichthys ﬂesus*) were grouped according to environmental similarities using *Hsc70* locus [22]. Pogson & Fevolden [23] suggested that a recent diversifying selection was reflected by the significant differentiation between coastal and Arctic populations of the Atlantic cod in northern Norway at the *PanI* locus. White et al. [24] also reported the differentiation between populations of the roundnose grenadier (*Coryphaenoides rupestris*) at locus *Crup7* was related to depth, suggesting that directional selection presumably acted on a linked locus. Several recent studies suggested directional differentiated microsatellites may have formed due to selection acting on their linked loci, which were related to environmental factors such as temperature, salinity and depth or water pressure [24-27]. As argued by Hemmer-Hansen et al. [22], adaptive divergence could be common in marine fishes.

The small yellow croaker, *Larimichthys polyactis* (Bleeker), is an important fishery species endemic to the Northwest Pacific, inhabiting coastal waters across the Bohai Sea, the Yellow Sea and the East China Sea [28]. As one of the most important fishery species in the Northeast Asian countries with annual catching production of more than 100 000 tons, *L. polyactis* are subjected to a high fishing pressure and now approaches overexploited [29-32]. This fish is an iteroparous species and mature at one year age with an average life span of about 20 years [33]. The average age of this species was at three years in 1960s and gradually decreased to one year at present due to high fishing pressure [29-33]. An understanding of the population structure and dynamics is critically urgent in order to conserve and manage this species. Studies based on spawning migration routes and morphological variation suggested three wild groups matched to three geographically well-separated spawning areas, the Bohai Sea and the northern Yellow Sea (BS-NYS), the central and southern Yellow Sea (CYS-SYS) and the northern and central East China Sea (NECS-CECS), respectively [33,34]. This fish species migrate from their overwintering aggregations to the spawning grounds in early April and begin spawning from late April in the coastal waters of NECS to June in the coastal waters of BS-NYS with a gradually spatial and temporal change [33]. After spawning, this fish feed in the nearby coastal waters. From September to October, all the fish migrate to their overwintering grounds [33]. This species was considered to migrate to natal sites to spawn [33,34]. The overwintering aggregations are admixtures of these three spawning groups, although they are relatively isolated [33]. Accordingly, the morphology and spawning migration behaviors for these three groups were considered to be discrepant. For example, individuals from different groups varied in body lengths and in the number of soft rays of dorsal fin; the spawning migration for these groups started at different time with the NECS-CECS group starting first and the BS-NYS group last; the optimal spawning temperature for these groups also varied, with the NECS-CECS group having the highest spawning temperature and the BS-NYS group the lowest [33,35]. After spawning, *L. polyactis* will be feeding in the nearby waters. Several studies on genetic structure of *L. polyactis* in our studied areas obtained inconsistent or even conflicting results. Although studies using RAPD and AFLP [36-38] all suggested differentiation into three groups, the partition of these three groups in each study was different. Study based on mitochondrial control region showed no significant genetic structure, suggesting panmixia in *L. polyactis* [39]. Such disagreement in the conclusions of the divergence and structure of *L. polyactis* has hindered the stock management and conservation to some extent. Considering the low sampling resolution in these studies (for example, the AFLP analysis only included four samples; the mtDNA study only focused on the Yellow Sea) [37-39], a more comprehensive study is needed as the results from former studies are insufficient.

In this study, we analyzed a large sample of *L. polyactis* across its distribution range along the Pacific coast of China using anonymous and known functional genes linked microsatellites, and mtDNA. Our purpose was to evaluate the levels and patterns of genetic variation and differentiation. Further, we aimed to find the interacting effects of historical and recent gene flow on shaping the population structure of *L. polyactis*. We also tried to detect the footprints of local adaptation considering large effective population size and varying environmental variables in the distribution areas of this species. Among the microsatellites, we detected evidence of local adaptation across the distribution range of *L. polyactis*. Using different marker types, we studied the patterns of population structure and disentangled the effects of demographic history, migration, genetic drift and natural selection from environmental factors on population structure of *L. polyactis*.

## Materials and Methods

### Ethics statement

The species is not protected by Chinese law or by any of the countries where and when sampling was performed. It is a commercially harvested species in China and the other Northeast Asian countries.

### Sampling and DNA extraction

**Figure 1:**
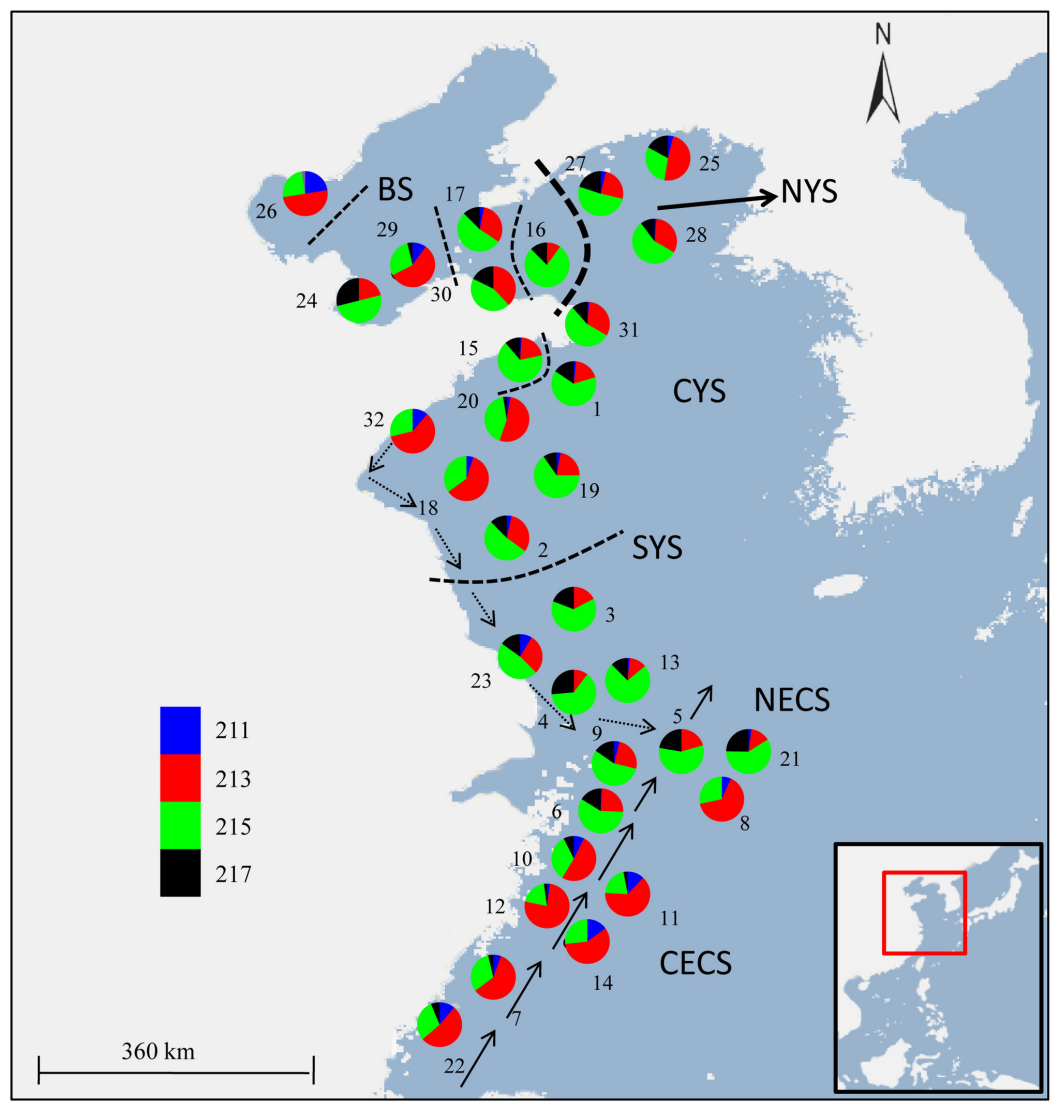
Map of sampling localities and allele frequencies at locus H16. Sample numbers match those in Table 1. Pie charts denote allele frequencies at H16, in which each color represents one allele identity. Well supported and less well supported barriers to gene flow based on neutral microsatellites are also shown with broken lines that are proportional to replicates support. The sea areas are shown in abbreviations corresponding to Table 1. The southward China Coastal Cold Current and northward Taiwan Warm Current are also shown with arrows.

Samples were collected from 32 localities (1104 individuals) along the coast of China across the species’ distribution range from 2007 to 2010 (Table 1; Figure 1). All the samples were collected by trawling method during fishery surveys performed by the Yellow Sea Fisheries Research Institute, Chinese Academy of Fishery Sciences. The animal work was approved by the Animal Care and Use Committee (IACUC) of the Yellow Sea Fisheries Research Institute. All the fishery surveys were approved by Ministry of Agriculture of the People’s Republic of China. Fish capturing and tissue sampling were all according to the Animal Care and Use Committee (IACUC) of the Yellow Sea Fisheries Research Institute. After trawling surveys, all the fish were dead. Muscle tissue or fin samples were obtained and preserved in 95% ethanol. Genomic DNA was isolated using either the standard phenol-chloroform extraction protocol or Promega Wizard Purification Kits (Promega, WI, USA). According to fisheries management, samples were partitioned into six geographical groups: BS, NYS, CYS, SYS, NECS and CECS. Individuals were identified based on morphological characteristics. Different strategies were employed in analyzing microsatellites and mtDNA sequences. For microsatellites, all 1104 individuals collected were genotyped. In mtDNA analysis, 395 individuals randomly selected from all these 32 samples were sequenced (Table 1). 

**Table 1:** Overview of *Larimichthys polyactis* samples used in this study and environmental factors at each locality.

Locality	N	Sampling date	Coordinates	Region	Salinity	Temp. 1	Temp. 2	Temp. 3	Temp. 4
24	19/5	August 3, 2007	37°20'N; 119°10'E	BS	NA	NA	NA	NA	NA
26	20/13	August 13, 2010	38°47'N; 118°33'E	BS	NA	NA	NA	NA	NA
29	40/14	August 12, 2010	37°45'N; 119°50'E	BS	NA	NA	NA	NA	NA
16*	20/10	May 4, 2009	37°50'N; 122°00'E	NYS	31.590	8.700	11.500	17.500	14.500
17*	32/12	May 6, 2009	38°10'N; 121°30'E	NYS	31.470	10.570	11.000	16.500	13.750
25	38/9	August 6, 2010	39°15'N; 123°45'E	NYS	NA	NA	NA	NA	NA
27	40/10	August 9, 2010	38°50'N; 122°51'E	NYS	NA	NA	NA	NA	NA
28	39/12	August 11, 2010	38°09'N; 123°23'E	NYS	NA	NA	NA	NA	NA
30	40/9	August 11, 2010	37°49'N; 121°47'E	NYS	NA	NA	NA	NA	NA
1	32/16	April 10, 2007	35°59'N; 122°30'E	CYS	32.190	9.250	NA	NA	NA
2	29/12	May 2, 2007	34°06'N; 121°12'E	CYS	31.320	14.560	NA	NA	NA
15*	49/14	May 3, 2009	36°25'N; 121°45'E	CYS	31.760	9.430	11.500	17.000	14.250
18	20/10	May 17, 2009	34°51'N; 120°50'E	CYS	30.080	8.470	NA	NA	NA
19	36/11	May 17, 2009	34°51'N; 121°49'E	CYS	31.290	8.390	NA	NA	NA
20	19/12	May 17, 2009	33°54'N; 121°07'E	CYS	29.980	8.160	NA	NA	NA
31	40/12	August 1, 2010	37°08'N; 122°52'E	CYS	NA	NA	NA	NA	NA
32	40/12	June 5, 2010	36°29'N; 119°51'E	CYS	NA	NA	NA	NA	NA
23*	47/16	April 30, 2010	32°15'N; 121°45'E	SYS	31.000	11.700	14.500	19.500	17.000
3	26/11	May 3, 2007	32°55'N; 122°33'E	SYS	32.520	11.740	NA	NA	NA
4*	34/11	May 7, 2007	31°48'N; 122°40'E	NECS	32.310	16.798	15.000	20.000	17.500
5	56/22	May 8, 2007	30°42'N; 123°37'E	NECS	33.820	17.710	NA	NA	NA
6*	53/23	May 10, 2007	30°04'N; 122°47'E	NECS	33.430	16.930	14.000	18.500	16.250
9*	26/11	May 15, 2008	30°24'N; 122°52'E	NECS	34.000	17.814	15.000	19.500	17.250
13	38/12	June 13, 2008	31°51'N; 122°41'E	NECS	33.252	13.862	NA	NA	NA
21	47/15	April 30, 2010	30°47'N; 124°50'E	NECS	33.638	14.000	NA	NA	NA
8	30/13	May 13, 2008	30°33'N; 124°02'E	NECS	33.252	16.802	NA	NA	NA
7*	27/12	May 13, 2007	27°50'N; 121°47'E	CECS	33.267	18.778	18.000	22.500	20.250
10*	40/10	May 16, 2008	29°23'N; 122°34'E	CECS	32.409	17.464	16.000	20.500	18.250
11	33/11	May 16, 2008	29°02'N; 123°07'E	CECS	33.861	20.219	NA	NA	NA
12*	24/10	May 17, 2008	28°34'N; 122°15'E	CECS	34.042	19.020	16.000	21.000	18.500
14	30/12	June 19, 2008	28°22'N; 122°35'E	CECS	33.213	19.420	NA	NA	NA
22*	40/13	April 28, 2010	27°43'N; 121°20'E	CECS	32.000	18.500	18.000	22.000	20.000

N, sample size (subjected to microsatellites⁄mtDNA DNA analysis); BS: the Bohai Sea; NYS: the northern Yellow Sea; CYS: the central Yellow Sea; SYS: the southern Yellow Sea; NECS: the northern East China Sea; CECS: the central East China Sea; * denotes coastal samples considered to be spawning when sampling; Temp. 1, 2, 3, 4 are temperature when sampling, average temperature in April, average temperature in May and average temperature in April and May, respectively; NA., not available.

### Microsatellites genotyping and mtDNA sequencing

Firstly, all sampled individuals were genotyped using 22 of our newly developed putatively neutral microsatellite loci, namely, H5, H16, H22, H26, H31, H33, H37, H38, H43, H44, H47, H51, H54, H65, H70, H71, H75, H80, H82, H84, H106 and H111 [40]. Besides, we employed six more microsatellites identified from functional genes including hot shock protein 27 (HSP27), growth hormone (GH), CC chemokine (CCC), growth differentiation factor-8 (GDF8), cytochrome P450 (P450) and hepatocellular carcinoma-associated antigen 127 (HCA127), and the microsatellites were located in the 3’UTR or 5’UTR of these genes (Table S1). These genes are well known to have functions in stress response, disease resistance, growth and development. We assumed that these genes could be under selection in the studied populations. These loci were firstly genotyped in several samples from different geographical regions to estimate the genetic differentiation. As population genetic studies suggest that loci of relative high level of divergence are likely to suffer from natural selection, we therefore further genotyped all the samples using the loci of higher level of differentiation. For the above loci, forward primers were 5′-labeled with a fluorescent dye HEX or 6-FAM. PCR amplification was also performed according to Molecular Ecology Resources Primer Development Consortium [40]. PCR products were separated on an ABI PRISM 3730 DNA automated sequencer (Applied Biosystems). Fragment size was measured according to the ROX-500 standard using GeneMapper (Applied Biosystems). 

Two mtDNA fragments of Cytochrome c Oxidase subunit I (*COI*) and Cytochrome b (*Cytb*) genes were targeted for sequencing using the following primer sets: 5ʹ-cgttataatcttctttatag-3ʹ and 5ʹ-gtgtcatgaaggacaatgtc-3ʹ for *COI*, and 5ʹ-gcctgatgaaactttggctc-3ʹ and 5ʹ-caaggactccgcctagtttg-3ʹ for Cyt*b*, respectively. PCR amplification was performed in a 50-μL volume containing 0.5μM of each primer, 0.2mM dNTP, 1.5mM MgCl_2_, 1×PCR buffer, 1U *Taq* DNA polymerase (Fermentas), and 20ng template genomic DNA. PCR program was as follows: 5 min at 94°C followed by 35 cycles of 30 s at 94°C, 30 s at 55°C, and 60 s at 72°C with a final extension of 5 min at 72°C. PCR products were then purified and used as templates for sequencing in both directions with primers above. Sequencing work was also conducted on an ABI PRISM 3730 DNA automated sequencer. Sequences have been deposited in GenBank with accession no.JN243355-JN243749 and JN250600-JN250994.

### Microsatellite DNA analysis

Genetic variation indices including the number of alleles (A), observed heterozygosities (H_O_), expected heterozygosities (H_E_), inbreeding coefficient (F_IS_) and allele richness (A_R_, an estimate of allelic diversity independent of sample size) were calculated in FSTAT version 2.9.3.2 [41]. Exact tests of Hardy-Weinberg equilibrium (HWE) in each sample for each locus and linkage disequilibrium (LD) between pairs of loci were tested using the Markov chain methods (10 000 dememorizations, 1000 batches, and 10 000 iterations per batch) performed in Genepop 4.0 [42,43]. Wright’s F statistics and global F_ST_ values with 95% confidence interval (CI) were also estimated using FSTAT version 2.9.3.2 [41]. The genotyping errors and the contribution of null alleles were checked by using Microchecker [44]. The statistical power to detect genetic divergence was measured for all the samples and markers using POWSIM 4.0 to evaluate the hypothesis of genetic homogeneity under Fisher’s exact tests [45]. Estimates of power were calculated as the proportion of significant outcomes in the 1000 times of simulations.

For microsatellites neutrality, we firstly used a Bayesian simulation-based test implemented in BAYESCAN [46] to test the microsatellites neutrality. This Bayesian method is based on the measures of locus-population specific F_ST_, which reflect contributions from locus-specific effects, such as selection, and population-specific effects such as genetic drift and immigration rates. The program BAYESCAN defines the population and locus specific effects on F_ST_ values and estimates its posterior probability using a reversible jump Markov chain Monte Carlo approach (MCMC). Furthermore, we used the method of Beaumont & Nichols [47] implemented in the program LOSITAN [48]. This method assumes that outlier loci under diversifying selection would show increased level of population differentiation and further compares the level of population differentiation correlated with heterozygosity at individual locus to simulated distribution of loci based on observed level of population differentiation. However, it has recently been demonstrated that the results of this method may be biased by the presence of strong hierarchical population structure and lead to increased level of false positives [49]. Therefore, we further performed the test by Excoffier et al. [49] that is based on the method of Beaumont & Nichols [47] under a hierarchical island model. Outlier loci detection was conducted in terms of F_ST_ across all populations and under a model of 10 groups each consisting of 50 populations. We performed this test based on 100 000 simulated loci and by using the software Arlequin 3.5 [50].

The population structure was assessed using pairwise F_ST_ matrices acquired by computing pairwise Wright’s F-statistics. In this analysis, we estimated two types of F_ST_ matrices: only the neutral loci and the outlier loci under diversifying selection detected in outlier tests. The significance of the tests were computed by a permutation with 10 000 replicates using ARLEQUIN 3.5 [50]. In order to compare the patterns of genetic structure between/among these marker types, Principal component analysis (PCA) based on gene frequency data was performed using PCAGEN [51]. We also analyzed the population structure for the neutral and the whole data sets using the program Structure 2.3.3 to visualize the genetic divergence among samples and locations [52]. This program was used to infer the number of putative clusters (K) and assign individuals into corresponding clusters. We performed this analysis 20 times under admixture model and using 10^5^ iterations after a 10^5^ burn-in length with K ranging from 1 to 8. The locations were included as priors considering rather low genetic divergence in this species. The most likely structures were evaluated using K and ΔK methods [53]. The effects of geographical isolation on the genetic structure were assessed in the form of isolation by distance (IBD) using Mantel tests in the program IBD [54,55]. The pairwise distance was estimated as the nearest marine distance between two collections using Google Maps. 

The population structure for neutral loci was also investigated using a landscape genetics method in the software BARRIER [56]. This program aims to identify the geographical breaks with genetic discontinuities in genetic structure between populations. The robustness of barriers was assessed by running BARRIER on 100 F_ST_ matrices, generated by bootstrapping over loci. As this landscape genetics approach highly favors a sufficient spatial resolution of sampling, it was considered well appropriate for our data sets.

In order to elucidate the effects of different environmental factors on population structuring, we used the hierarchical Bayesian approach of Foll and Gaggiotti [57], implemented in the program GESTE. This method estimates the specific F_ST_ values for each population and then relates them to environmental factors using a generalized linear model. In our study, we assessed seven environmental variables including sampling temperature and salinity, latitude and longitude, as well as historical average temperature in April, in May and in April and May retrieved from weather.sina.com.cn (shown in Table 1), which leads to 2^7^ (= 128) alternative regression models. This method calculates the posterior probabilities for each of these models using a reversible jump MCMC approach. We performed this analysis under default parameters. Based on the results of GESTE, we also tested the level of correlation between pairwise F_ST_ and pairwise differences of environmental factors of the highest posterior probability in outlier tests using Mantel test. As the environmental factors above are considered highly related to the behavior of spawning migration for marine fishes [58-61], the Mantel test was only performed for the available coastal samples, which are much more likely in the spawning status.

Demographic history of population size variations was investigated in the form of heterozygote excess based on neutral loci using the program Bottleneck version 1.2.02 [62]. Because the loss of rare alleles is much more rapid than common alleles in populations experiencing bottlenecks, populations experiencing recent size reduction would show heterozygote excess compared to the populations at equilibrium with the same number of alleles [63]. This analysis was performed under two-phase model (TPM, with 90% stepwise-mutation) with 1000 iterations and using the mode-shift test by Luikart et al. [64].

In outlier tests, we detected one anonymous locus, H16 showed much higher level of genetic divergence (see results). Blast against GenBank revealed that this locus showed more than 90% of sequence identity with the intron sequence of BAH domain and coiled-coil containing protein 1 (BAHCC1) gene of European seabass (*Dicentrarchus labrax*). Based on sequence homology among European seabass (GenBank accession no.CBN81236), zebrafish (*Danio rerio*, GenBank accession no.XP001334285) and orange-spotted grouper (unpublished data from genome sequencing program of our laboratory), we designed primers to amplify the corresponding sequence and aimed to confirm that H16 locus is also linked with BAHCC1 gene in *L. polyactis*. PCR was conducted using Takara LA kit ver. 2.1. Amplified fragments were ligated into PMD-18T vector (Takara) after purification using the E.Z.N.A. Gel Extraction Kit (Omega, USA) and were further sequenced in both directions.

### Mitochondrial DNA analysis

DNA sequences were edited and aligned using BioEdit version 7.0.5.3 [65] and DnaSP 4.10.4 [66]. Summary statistics of genetic diversity including haplotype diversity (h) and nucleotide diversity (π) were calculated using ARLEQUIN 3.5 [50].

Levels of genetic differentiation were estimated using pairwise Φ_ST_ computed in ARLEQUIN 3.5 [50]. Recent gene flow between populations was also estimated in the form of isolation by distance (IBD) using Mantel test in the program IBD [54,55].

Phylogenetic analysis of sequences was performed by maximum likelihood using TREEFINDER [67] and by Bayesian method using MrBayes 3.1.2 [68]. Before these two analyses, the most appropriate evolutionary model (GTR+I+G) was estimated with Akaike information criterion (AIC) in the program ModelTest 3.7 [69]. For the Bayesian method, we performed both partitioned and non-partitioned analysis. The partitioned analysis was executed according to the method of Wang et al. [70]. This strategy was used to eliminate the effect of incompatibility of sequences combination. In this analysis, we ran four independent chains for 2 × 10^6^ generations with a burn-in period of 10^6^. However, we could not obtain an average standard deviation of split value of less than 0.100 as suggested [68].

Historical population expansion was examined using Tajima’s D [71] and Fu’s F_S_ [72] statistics with 10 000 permutations calculated in ARLEQUIN 3.5 [50]. Demographic history was also tested using mismatch distribution. Populations experiencing recent expansion showed smooth or unimodal distributions, whereas demographically stable populations presented with ragged or multimodal distribution patterns [73]. Statistical significance was tested using the sum of squared deviation (SSD) and Raggedness index (R). Subsequently, we estimated the time of expansion in populations detected to have experienced expansion using the equation τ=2ut, where τ was the time of expansion expressed in the form of mutational units, u was the mutation rate per generation, and t was the time measured in generation since expansion. Mutation rates between 1% and 3% per million years for mitochondrial genes were selected according to Cantatore et al. [74]. The time of initial maturity for *L. polyactis* was estimated as one year according to Lin [35].

## Results

### Genetic variation

For all samples and loci combinations, there was little evidence of LD after correction for multiple comparisons. Exact tests showed no consistent deviations from HWE after sequential Bonferroni correction at the significance level of 0.05 (Table S2). MicroChecker found obvious evidence of null alleles at three loci, H22, H84 and P450. A summary statistics of genetic diversity for each sample per locus was shown in Table S2. No geographical pattern was observed in the distribution of A_R_ and heterozygosity across sampling localities (P > 0.05, Mann-Whitney U test; Table S3). As the presence of null alleles may bias the results of outlier tests and population differentiation, we therefore conducted further analysis excluding these three loci. The statistical power estimated by POWSIM [45] based on all the samples and applicable markers revealed that it was adequate to detect a low level of genetic divergence at F_ST_ = 0.001 with a probability of more than 0.9. 

Mitochondrial DNA sequence alignment comprised of 820 bp from *COI* and 692 bp from Cyt*b* and showed no evidence of stop codons and indels. Of the entire 1512 bp sequence, 208 sites were variable with 158 parsimony informative sites. We identified 278 haplotypes among the sequences of 395 individuals of *L. polyactis*. Haplotype diversity (h) and nucleotide diversity (π) showed no differences among geographical groups but significant homogeneity across samples (Table S3).

### Neutrality tests

**Figure 2:**
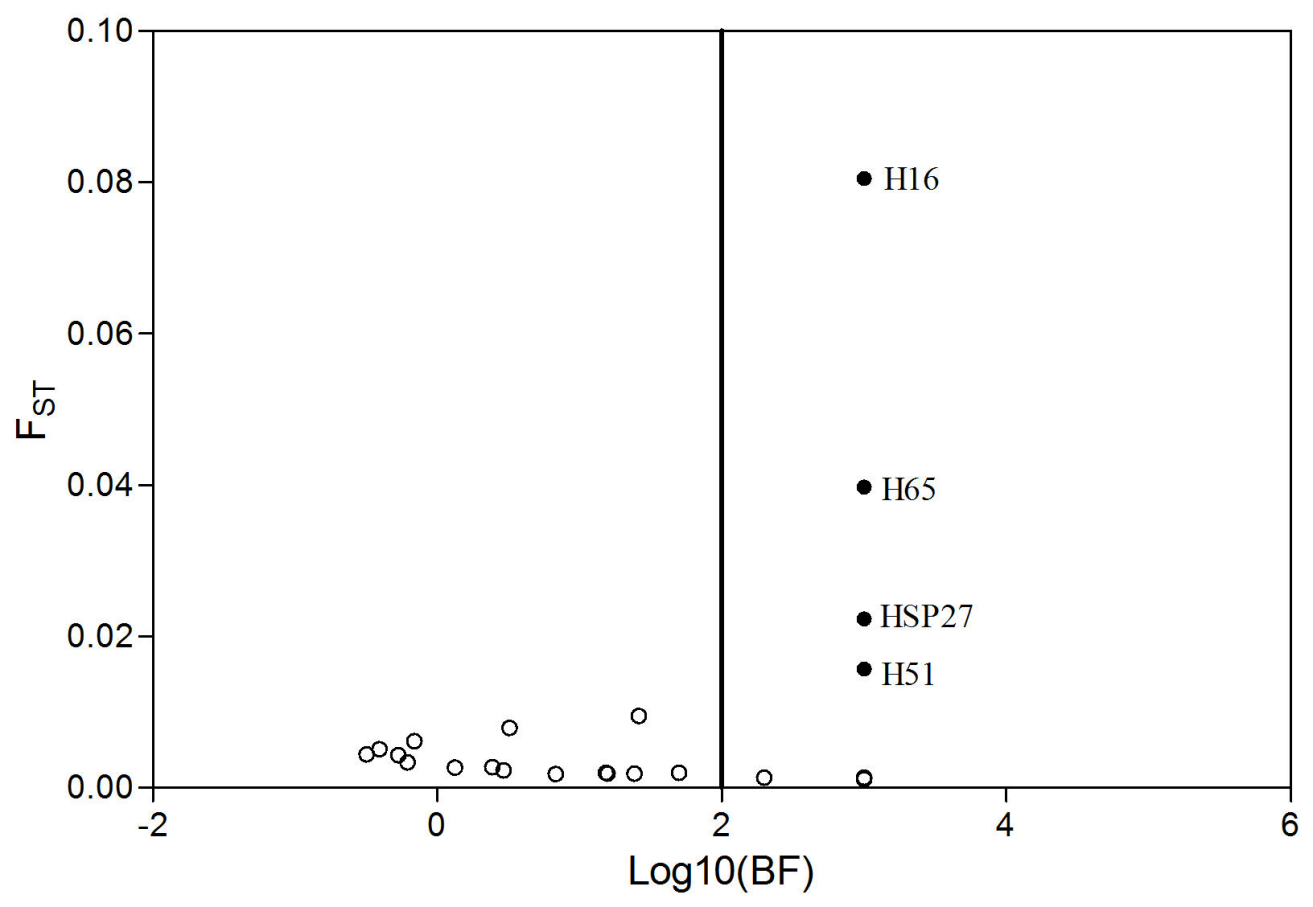
Results of the outlier tests using BAYESCAN. Loci identified as significant outliers under the decisive criterion of Jeffrey’s interpretation are denoted as solid circles.

**Figure 3:**
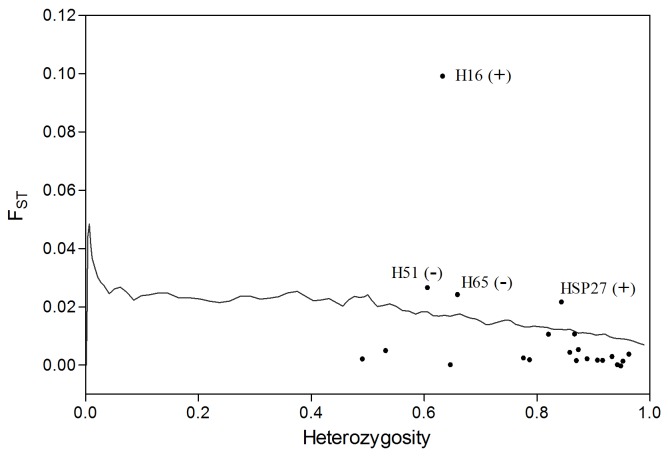
Results of the outlier tests using LOSITAN (outliers are above the 99% confidence interval curve) and under hierarchical island model using Arlequin (H16 and HSP27 are still outliers, whereas H51 and H65 lost significance as outliers).

The overall F_ST_ for whole data set was 0.008 (95% CI: 0.003 - 0.016) and was highly significant (P < 0.001). However, the overall F_ST_ value for locus H16 (F_ST_ = 0.092) was much higher than the other loci, indicating diversifying selection at this locus. Outlier tests based on BAYESCAN showed that four loci H16, H65, HSP27 and H51 were under diversifying selection under the decisive criterion of Jeffrey’s interpretation that loci with Log _10_ (Bayes factors) > 2 were considered as outliers (Figure 2) [46]. Using the program LOSITAN, the above four loci were also suggested to be outliers over 99% confidence interval (Figure 3). Under hierarchical island model, only three loci H16, H65 and HSP27 were outliers at the significant level of 0.05. After Bonferroni correction for multiple comparisons, however, H65 lost the significance to be outlier (Figure 3). In total, the consistent results among these outlier tests suggest that H16 and HSP27 are candidate loci under diversifying selection. 

### Population structure

**Figure 4:**
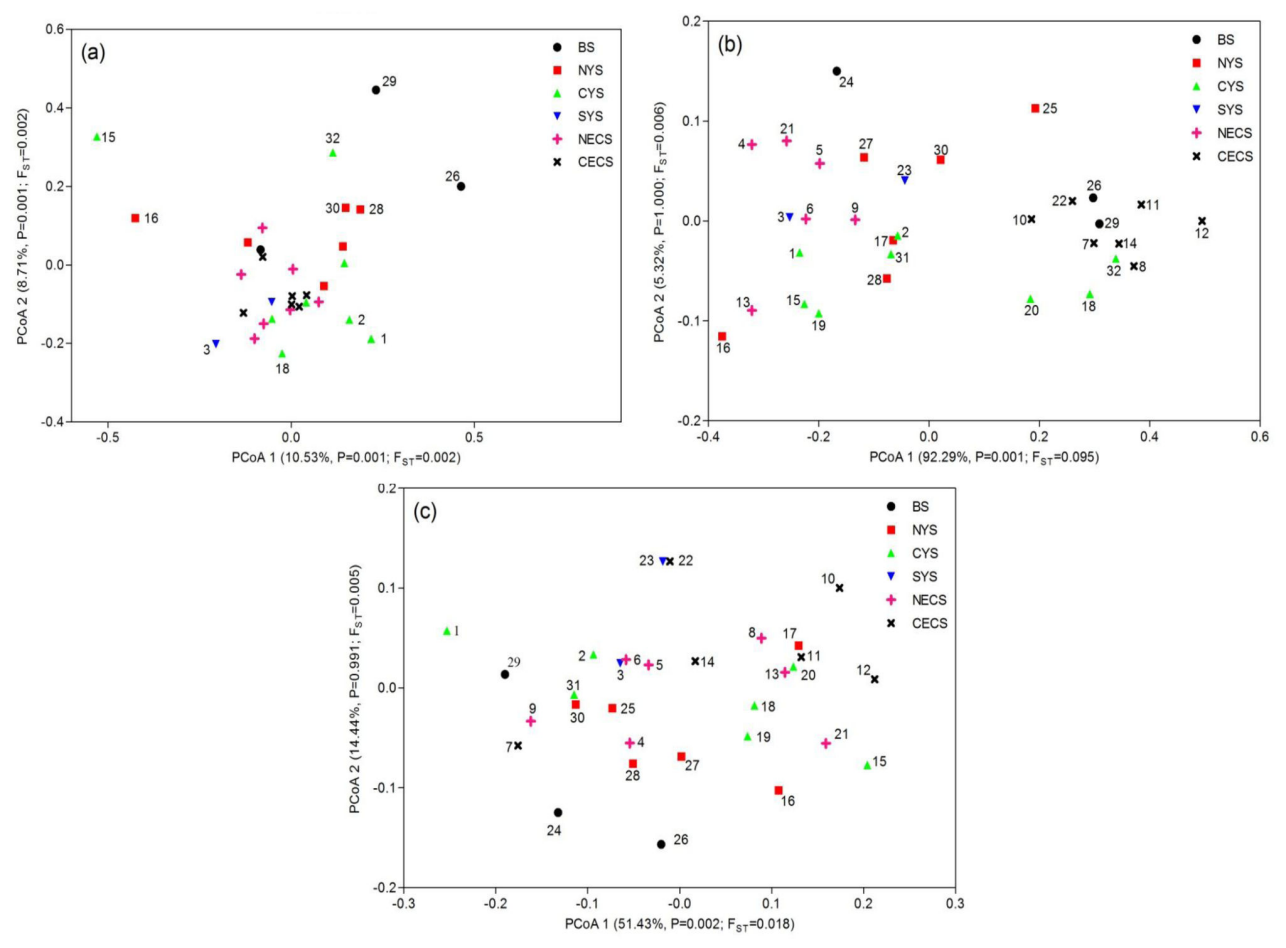
PCA plotting of population differentiation based on (a) neutral microsatellites; (b) locus H16; (c) locus HSP27.

The overall neutral F_ST_ was 0.004 (95% CI: 0.002 - 0.007) and was statistically significant (P < 0.001). Nevertheless, this value was markedly lower than that of the directional selected loci H16 (F_ST_ = 0.092, P < 0.001) and HSP27 (F_ST_ = 0.020, P < 0.001). In PCA plotting, we didn’t observe a clear geographical pattern of population structure based on neutral loci (Figure 4a). The significant genetic differentiation was mainly caused by the genetic heterogeneity of six samples (26, 29, 16, 15, 32 and 3) from the Bohai Sea and the northern Yellow Sea (Figure 4a and Table S4a). Besides, pairwise F_ST_ analysis revealed the southernmost sample 22 was slightly divergent from several other samples (Table S4a). Excluding this sample, we detected little evidence of genetic differentiation among the samples in the East China Sea (Table S4a), suggesting panmixia of *L. polyactis* in this distribution region. We also detected a weak pattern of IBD for the neutral loci across all the samples (R = 0.108, P = 0.022).

On the contrary, the genetic divergence for both loci under diversifying selection not only showed much higher values but also presented significant geographical patterns. In detail, pairwise F_ST_ analysis at locus H16 revealed a clear separation between samples from NECS and CECS (Table S4b). PCA plotting for H16 showed two clearly differentiated groups among all samples and samples from NECS and CECS belonged to these two differentiated groups, respectively (Figure 4b). Moreover, allele frequencies at H16 exhibited significant differences between the two groups identified above, that allele 211 and 213 appeared more frequent in the ‘CECS group’ while allele 215 and 217 were richer in the ‘NECS group’ (Figure 1). However, we didn’t detect a pattern of IBD at locus H16 (R = 0.072, P = 0.100). Although we didn’t observe a clear population differentiation pattern at HSP27, we found several samples significantly differentiated from the other samples, which was different from the neutral loci (Figure 4c & Table S4c). The population differentiation at HSP27 also rejected the IBD pattern (R = 0.055, P = 0.134). 

**Figure 5:**
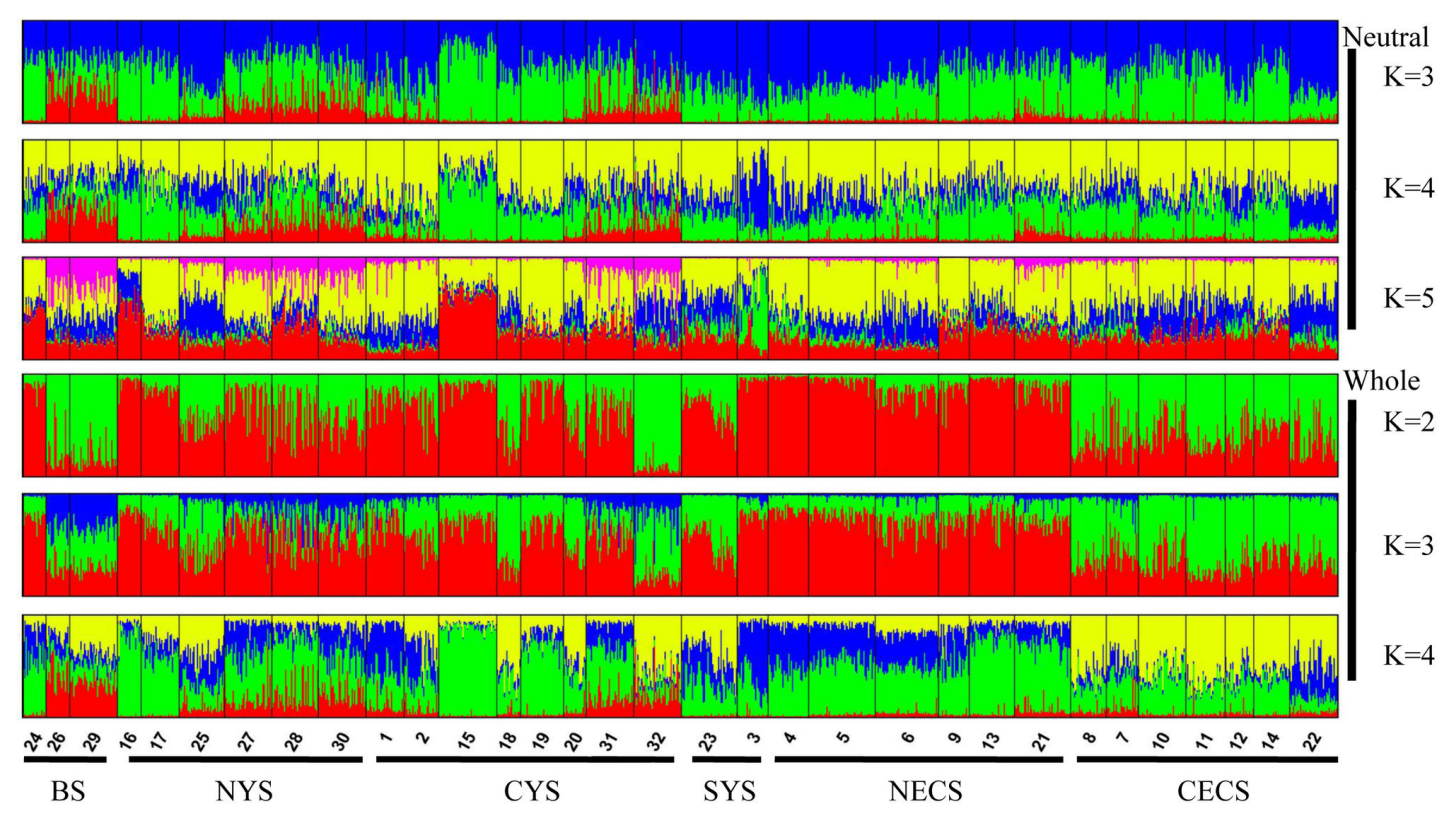
Plotting results of the program STRUCTURE for neutral loci with K ranging from 3 to 5 and for whole data set with K from 2 to 4, respectively. Sample identities and its corresponding geographical regions are also showed.

In the analysis of STRUCTURE, we determined the most likely genetic clusters using ΔK methods [53]. We didn’t obtain obvious K values due to low level of population differentiation. It was suggested that there were two clusters for the whole data set, whereas the number of clusters for the neutral loci was still unclear lacking of genetic heterogeneity (Figure S1). Nevertheless, we found some evidence of genetic heterogeneity at several samples including sample 26, 29, 27, 28, 30, 31 and 32 for neutral data set with ΔK values ranging from 3 to 4 (Figure 5). For the whole data set, we detected significant genetic heterogeneity across all the samples and the pattern of genetic structure was consistent with the results of F_ST_ and PCA analyses (Figure 5). 

The landscape genetics program BARRIER identified one well supported genetic discontinuity, which was located between the Bohai Sea and the Yellow Sea and isolated the samples of the Bohai Sea from the others (Figure 1). Less well supported genetic barriers among the samples of the BS and western NYS were also detected (Figure 1). Apart from these barriers above, there were also less well supported genetic separations that were placed in the CYS and SYS (Figure 1).

The results of the program GESTE were shown in Table 2 and provided suggestions on the environmental factors shaping the population structure. The locus H16 presented the highest posterior probabilities with relation to average temperature in May (Table 2). Mantel tests also revealed that the average temperature in May was positively associated with the population structure (R = 0.301, P = 0.013). However, we found little evidence that the studied environmental variables including temperature, salinity, longitude and latitude markedly influenced the population structure for the neutral loci and for the HSP27 (Table 2). 

**Table 2:** Sum of posterior probabilities of models that include a given factor.

Factors	Sum of posterior probabilites		
	H16	HSP27	Neutral loci
Sampling temperature	0.255	0.223	0.185
Average temperature in April	0.437	0.197	0.169
Average temperature in May	**0.518**	0.202	0.162
Average temperature in April & May	0.440	0.211	0.182
Spawning salinity	0.167	0.136	0.104
Latitude	0.317	0.187	0.157
Longitude	0.161	0.110	0.091

Factor with the highest score and probability is highlighted with bold.

We sequenced a genomic fragment of approximately 18 000bp by designing primers according to sequence homology among European seabass, zebrafish and orange-spotted grouper (Table S5). This sequence has been submitted to GenBank with accession no.JX027377. Further analysis revealed that this sequence had a similarity of more than 70% with the BAHCC1 genes of European seabass (accession no.CBN81236.1) and orange-spotted grouper (unpublished data). Locus H16 was located in the second intron of this gene. Analysis of the protein coding sequence showed that BAHCC1 gene was highly conserved among zebrafish, mouse (*Mus musculus*), human (*Homo sapiens*) and some other species (Figure S2). 

For mtDNA, pairwise Φ_ST_ analysis revealed no significant differentiation across all samples after correction for multiple comparisons (Table S4d). Phylogenetic tree presented only one lineage (Figure S3). Bayesian method that failed in attempts to construct trees also suggests only one lineage for mitochondrial haplotypes.

### Demographic history

Tests of heterozygote excess relative to equilibrium mutation drift expectations for microsatellites showed no evidence of recent bottleneck for all 32 samples. In mtDNA analysis, Tajima’s D and Fu’s F_S_ all showed significantly negative values in all group cases, strongly supporting the model of population expansion (Table S6). Mismatch distribution analysis revealed significant population expansion according to the consistent results of SSD and R statistics (Table S6). In total, microsatellites and mtDNA showed the same results regarding to population expansion. We used τ value (τ = 5.807, 95 % CI: 5.045 - 6.369) for all samples to calculate the expansion time because time since expansion (τ) showed no significant differences among groups. The estimated expansion time began at the period from 64ka to 192ka before present (BP).

## Discussion

### Evidence for diversifying selection

Identifying loci under possible selection using outlier tests must be critically assessed due to false positives [75]. Nevertheless, there are still some uncertainties about the results of these outlier tests due to the assumed models in the tests, demographic history of tested populations and different mutation rates among loci [75]. One solution to these uncertainties is to apply conceptually different tests and compare the results [75-77]. Here, we used conceptually different tests to identify the footprints of selection in *L. polyactis* employing microsatellite loci.

In our study, BAYESCAN and LOSITAN methods consistently suggested four loci including H16, H51, H65 and HSP27 as significant outliers. However, the hierarchical island model rejected locus H51 as an outlier. It should be noted that H65 also lost its significance of being as outlier under this model after correction for multiple comparisons. As argued by Hansen et al. [75], the application of multiple outlier tests would also undoubtedly lead to a number of false positives. For this reason, we took strict measures and only treated locus H16 and HSP27 as possible outliers for further analysis. 

 The outlier status of H16 under diversifying selection was further supported by its rather higher F_ST_ value, the significant association of population structure with environmental factors at the spatial scale and also by rejection of isolation by distance model. In contrast, the putatively neutral loci presented rather lower genetic differentiation and also a pattern of isolation by distance, which is typical of neutral mutations in high gene-flow marine fish species [14,22]. As suggested by Vasemagi & Primmer [76], such association between allele frequency and environmental variables can be viewed as evidence for directional selection affecting the particular locus. Overall, the results above strongly suggest that natural selection is working directly on this locus or by hitchhiking in *L. polyactis*.

It is potentially difficult to determine the selective agents for adaptive divergence. However, the effects of environmental factors on population differentiation of *L. polyactis* may provide important clues. In the analysis of GESTE, the genetic mutations at H16 showed higher level of associations with average month temperature during spawning season especially in May, supported by Mantel tests. These results consistently indicate the genetic differentiation at H16 correlates with temperature during spawning season. Previous study reported significant temperature differences between the NECS and CECS and samples between these two areas showed a different preference to temperature at the spawning time [78]. In our dataset, we also found that samples between these two areas were clearly isolated and presented correlations to temperature differences. Therefore, temperature could be the most important selective agent driving adaptive differentiation regarding to locus H16.

In this study, we found H16 to be located in the introns of the BAHCC1 gene in *L. polyactis*. BAHCC1 protein plays important functions in regulating embryonic development and growth in multi-cellular eukaryotes [82-84]. Several studies revealed that BAHCC1 mutations and expression profiles were associated with cytoskeleton and rapidly growing cells and organisms. It is possible that the genetic divergence at BAHCC1 locus in this study is related to different growth rates and cytoskeleton strength of the *L. polyactis* from different geographical regions. This information is important for us to unveil the mechanism of adaptive divergence at this locus, although further functional studies on this gene are needed. 

In this study, we also found evidence of diversifying selection on outlier heat shock protein locus, HSP27, a protein mainly involved in regulating stress responses. HSP27 showed a higher level of genetic divergence and also different population structure pattern compared to putative neutral loci. Although no association of HSP27 with temperature was detected, this environmental factor cannot be ruled out as a potential selective agent. It is likely that as a selective agent, temperature is temporally different from that of the spawning season, therefore showed no associations with genetic differentiation in this study. In addition, heat shock protein genes were found to be expression responsive to different salinity for marine fishes [82]. Besides, these genes also have roles in immune responses and in fighting against environmental pollution [22,83,84]. Along the China coast of the Bohai Sea, the Yellow Sea and the East China Sea, there are plenty of microenvironments, where the temperature, salinity, the levels of pollution and pathogen conditions are largely different. Firstly, the interactions among the ocean currents, coastal currents and the fresh waters flowing in would definitely cause different temperature and salinity everywhere [85]. Secondly, due to industrial unbalance among different coastal cities, the severity of seawater pollution is also different among regions. These environmental factors above, in addition to some other factors of regional differences would lead to different pathogen conditions. In total, these environmental variables may jointly contribute to the adaptive divergence at HSP27 locus in *L. polyactis*.

 Our results here likely indicate that diversifying selection in *L. polyactis* is common in the current distribution areas. Recently, several studies have found the evidence of local selection in marine fishes, such as the Atlantic cod, European ﬂounder, killifish (*Fundulus heteroclitus*), and so on [14], suggesting local adaptation is common in marine fish species.

### Patterns of population structure

The population structuring of marine fishes has been considered to be the results of interaction among environmental factors, behaviors, genetic drift and demographic history. In our work, we provided different and comprehensive insights into the genetic structuring of *L. polyactis* using putatively neutral and diversifying selected microsatellites and mtDNA analysis. The joint application of these different marker types not only disentangled the effects of different factors shaping the genetic structure, but also suggested unique evolutionary history of *L. polyactis*.

Multiple allopatric glacial refugia are necessary for population divergence during Pleistocene [86]. In our study, we detected only one lineage of *L. polyactis* across distribution range using mtDNA, suggesting only one refuge for *L. polyactis* during Pleistocene glaciations, the ancient East China Sea. The Bohai Sea and the Yellow Sea were exposed due to low sea level during this period. Considering the long-term glacial period and frequent interglacial periods, immediate gene flow during this period might have blurred the existence of multiple glacial refugia. However, phylogenetic studies on marine species in the studied areas found multiple lineages coexisting in the current Yellow Sea and East China Sea and no evidence of interglacial gene flows blurring genetic lineage divergence [12,70]. In this case, the current population structure of *L. polyactis* could have been shaped by relatively recent events but not by glacial events.

In our study, inconsistent results between neutral and diversifying selected microsatellites reflect the interaction effects of recent gene flow or migration, genetic drift and local adaptation on population structure. Based on neutral data, we found high gene flow and weak population structure, as well as a weak pattern of IBD across samples. Nevertheless, the landscape approach might provide some evidence of geographical structure, which reflects the effects of migration routes on population structure. On one hand, the program BARRIER identified one strong genetic discontinuity being placed between the BS and the NYS. This barrier shows little difference with the boundary between the northern and the central migratory groups partitioned by fishery research based on migration routes [33,34]. The genetic isolation of croakers between the two seas was most likely caused by the narrow Bohai straight and the islands in the straight. On the other hand, the less well supported genetic barrier in the SYS is consistent with the partition between the southern and the central migratory groups. In total, BARRIER analysis may have detected the boundaries among three migratory groups of *L. polyactis*. Additionally, several less well supported genetic barriers were also identified in the Bohai Sea and the northern Yellow Sea, suggesting high genetic heterogeneity for the populations in these areas. Importantly, we detected some evidence of panmixia for *L. polyactis* in the East China Sea, revealing that *L. polyactis* in this area is an independent migratory group as suggested by Liu [33] and Ikeda [34].

Many studies have revealed population structuring in migratory marine fishes such as weakfish (*Cynoscion regalis*), Atlantic herring (*Clupea harengus*) and Atlantic Bluefin Tuna (*Thunnus thynnus*), indicating the influences of migration routes on population structuring by blocking gene flow [5,9,87]. The low dispersibility of larvae and juveniles due to larvae localization and a short larvae period may be an important factor hindering gene flow among different migratory groups. Transport between genetically different groups is more likely for larvae and juveniles than adults, because migrating adults have a lower chance of joining new habitats compared to larvae and juveniles [88-90]. The early life history of marine fishes may be a good marker for the dispersibility of larvae and juveniles [91]. *L. polyactis* spawn eggs typically at waters near shore and juveniles are also prone to live in inshore waters and shallow estuaries [28], suggesting limited dispersibility for the larvae and juveniles [12]. However, this assumption requires the support of tracing data.

In addition to high gene flow and weak population structure for neutral differentiation, we detected strong population structure using loci under diversifying selection, especially H16. Based on the data of H16, we identified two genetically differentiated groups across samples, with samples belonging to each group clearly isolated in the two contiguous areas of the NECS and CECS. However, the other samples from these two differentiated groups coexisted in the Bohai Sea and the Yellow Sea. All these results suggest that the BS, NYS and CYS are likely the second contact zone of samples from these two differentiated groups distributed in the NECS and CECS. Additionally, the differentiation at this locus showed evidence for adaptation to temperature variations during spawning season as suggested by the program GESTE and Mantel tests. Together, the different patterns of population structuring between neutral loci and loci under diversifying selection demonstrate that the strength of local selection is much higher than genetic drift and migration even in a high gene flow background for *L. polyactis*.

### Insights for evolution history

During the last glacial maximum (LGM), the ancient East China Sea was lowered into a trough due to the low sea level and the shallow shelf [92,93], which explains the single mitochondrial phylogenetic lineage in *L. polyactis*. The initial expansion of *L. polyactis* began after LGM, when the Yellow Sea and the Bohai Sea appeared after 19ka BP (before present) and the ocean currents in studied areas began to form since then [94]. The southward China Coastal Cold Current and the northward Taiwan Warm Current intersected in the East China Sea between the current NECS and CECS [95]. These two ocean currents caused significant differences in environmental factors between the NECS and CECS, such as temperature, salinity and bait [85]. Among these factors, temperature is likely the strongest selective agent driving *L. polyactis* differentiation because the physiological activities of migratory marine fishes are very sensitive to temperature changes [60]. Seen from the differentiation pattern of H16, *L. polyactis* may have initially differentiated under selection of temperature between NECS and CECS, and then recolonized the Bohai Sea and the Yellow Sea. As a result, these two seas seemed like the secondary contact zone for *L. polyactis*. However, the mechanism of maintaining such pattern of divergence was still unclear. The adaptive differentiation for samples in the Bohai Sea and the Yellow Sea may have resulted from the physiological behavior of native environments adaptation and the higher strength of selection than the migration rates [17].

As the formation of the southward China Coastal Cold Current was after 14ka BP [96], it is unlikely for the adaptive differentiation at H16 to have occurred prior to this event. The formation of the Yellow Sea Warm Current at about 6ka BP may have driven *L. polyactis* recolonizing the Yellow Sea and the Bohai Sea by providing suitable conditions [96]. This expansion was also supported by neutral mutation that samples of the BS and the Yellow Sea showed high level of genetic heterogeneity as suggested by F_ST_ and BARRIER analyses.

Further, the modern migration routes of *L. polyactis* likely have formed after recolonizing the Bohai Sea and the Yellow Sea at about 6ka BP. As this period was sufficient to built up significant genetic differentiation considering the relatively short generation time of *L. polyactis* (about one year), the low differentiation for neutral microsatellites is most likely due to high effective migration rates rather than genetic drift [10]. In addition, the population structuring exhibited a weak pattern of IBD for neutral loci, which also indicates migration plays an important role in contributing spatial genetic structure [97].

### Implications for conservation and management

Effective stock management and conservation are hindered by the lacking of knowledge of the demographic history, the patterns of gene flow and the adaptation to environmental factors [98]. In particular, an understanding of the relationship between adaptive divergence and environmental factors can help genetic stock identification and prediction of the adaptive responses to future environmental changes, which is critical to the management of marine fish species of large effective population size [3,98]. In our study, we identified genetically and demographically independent units and also stock boundaries using different types of genetic markers. In population genetic studies, the directional selected markers with known functions are preferred [99]. In this study, we presented important evidence for the selection pressure on *L. polyactis* differentiation from environmental factors, which can help understand the adaptive evolutionary processes [100]. Last but not least, studies on the neutral loci showed a higher level of genetic heterogeneity in the samples in the Bohai Sea and the Yellow Sea than samples in the other distribution areas. The discussion above provides important information for stock management and conservation and for the future maintenance of the population dynamics and evolutionary processes of *L. polyactis*.

## Conclusions

Our work demonstrates that joint application of different genetic marker types to study population dynamics provides a comprehensive understanding of the interplay among different evolutionary forces. Our results showed that different patterns of population structure using mitochondrial DNA, putatively neutral and outlier microsatellites disentangle the effects of demographic history, migration, genetic drift and environmental factors on the population divergence of *L. polyactis*. At the same time, the adaptive differentiation provides novel insights into the population structure and evolutionary process of *L. polyactis* and serves as important information for designating stock boundaries and evolutionary units for *L. polyactis* management and conservation. Although the mechanism of local adaptation is currently unclear, our study adds to the understanding of the evolutionary pattern of migratory marine fish species, which can help predict future variation of biodiversity and establish conservation strategies.

## Supporting Information

Table S1 **Summary of the microsatellites linked to functional genes.**
(DOCX)

Table S2 **Summary statistics at 25 microsatellite loci across 32 samples of *Larimichthys polyactis*.**
(XLSX)

Table S3 **Genetic diversity of microsatellites and mitochondrial DNA for each sample.**
(DOCX)

Table S4 **Values for pairwise differentiation (below diagonal) and associated P values (above diagonal).** (a) F_ST_ values based on 20 neutral microsatellites loci. (b) F_ST_ values based on locus H16. (c) F_ST_ values based on locus HSP27. (d) Φ_ST_ values based on mtDNA. Differentiation with P < 0.0001 (correction for multiple tests of 496 comparisons at 0.05 levels) was highlighted in bold. Inter-population differentiation is highlighted with light shading.
(XLSX)

Table S5 **Primer sequences used for BAHCC1 gene cloning.**
(DOCX)

Table S6 **Demographic statistics for mitochondrial DNA.**
(DOCX)

Table S7 **Sampling location and Genebank accession number for each sample.**
(DOCX)

Figure S1 **ΔK values calculated according to Evanno et al.** **(2005) for 20 and 22 loci data sets, respectively**.
(DOCX)

Figure S2 **Sequence alignment of BAHCC1 protein sequences among several species.**
(DOCX)

Figure S3 **Phylogenetic tree of the small yellow croaker (*Larimichthys polyactis*) based on mitochondrial DNA haplotypes.** Bootstrap supports of more than 50% from 1000 replicates are shown. The Large yellow croaker (*Larimichthys crocea*) is used as outgroup (GenBank no. EU339149).
(DOCX)
