# Supplementary material for: Population Genetic Studies Revealed Local Adaptation in a High Gene-Flow Marine Fish, the Small Yellow Croaker (Larimichthys polyactis)
Source: PLoS One. 2013 Dec 12;8(12):e83493. doi: 10.1371/journal.pone.0083493 (PMC3861527; doi:10.1371/journal.pone.0083493)
Supplement: Table S1 — Summary of the microsatellites linked to functional genes. (DOCX) [file pone.0083493.s001.docx]

**Table S1** Summary of the microsatellites linked to functional genes.

| Locus | Tann. | GenBank no. | Position | Sequence (5'-3') | |
| --- | --- | --- | --- | --- | --- |
| hot shock protein 27 (HSP27) | 56°C | HQ651057 | 3'UTR | F: | tcaacgtggacacacaaggt |
|  |  |  |  | R: | ctggggttggctcattaccag |
| growth hormone (GH) | 56°C | AY090592 | 3'UTR | F: | tttgatgctctgctgtctgc |
|  |  |  |  | R: | tgttgagttgacgctgttcc |
| CC chemokine (CCC) | 56°C | HQ540430 | 3'UTR | F: | tccctcctctccatcatcac |
|  |  |  |  | R: | tcgtcaaaaaggcattgaaa |
| growth differentiation factor-8 (GDF8) | 56°C | AY842933 | 3'UTR | F: | ccttcacaaccggcataact |
|  |  |  |  | R: | tgttggctacgagtggagtg |
| cytochrome P450 (P450) | 56°C | EU169141 | 5'UTR | F: | ggaacaagtctgggggagtt |
|  |  |  |  | R: | ccagcacaacctggagaagt |
| hepatocellular carcinoma-associated | 56°C | JF440968 | 5'UTR | F: | gccggctattgtgttttgat |
| antigen 127 (HCA127) |  |  |  | R: | actccagagcctcgaactca |
