# Supplementary material for: Population Genetic Studies Revealed Local Adaptation in a High Gene-Flow Marine Fish, the Small Yellow Croaker (Larimichthys polyactis)
Source: PLoS One. 2013 Dec 12;8(12):e83493. doi: 10.1371/journal.pone.0083493 (PMC3861527; doi:10.1371/journal.pone.0083493)
Supplement: Table S3 — Genetic diversity of microsatellites and mitochondrial DNA for each sample. (DOCX) [file pone.0083493.s003.docx]

**Table S3** Genetic diversity of microsatellites and mitochondrial DNA for each sample.

| Sample | n | A_R_ | H_O_ | H_E_ | N | h ± SD | π ± SD |
| --- | --- | --- | --- | --- | --- | --- | --- |
| 24 | 19/5 | 10.404 | 0.755 | 0.806 | 5 | 1.000±0.127 | 0.002±0.002 |
| 26 | 20/13 | 10.330 | 0.738 | 0.798 | 13 | 1.000±0.030 | 0.004±0.002 |
| 29 | 40/14 | 10.834 | 0.742 | 0.807 | 13 | 1.000±0.027 | 0.004±0.002 |
| 16 | 20/10 | 10.363 | 0.774 | 0.806 | 10 | 1.000±0.045 | 0.003±0.002 |
| 17 | 32/12 | 10.745 | 0.774 | 0.810 | 12 | 1.000±0.034 | 0.004±0.002 |
| 25 | 38/9 | 10.667 | 0.759 | 0.803 | 9 | 1.000±0.052 | 0.003±0.002 |
| 27 | 40/10 | 10.762 | 0.749 | 0.821 | 10 | 1.000±0.045 | 0.004±0.002 |
| 28 | 39/12 | 10.698 | 0.745 | 0.808 | 11 | 1.000±0.034 | 0.004±0.002 |
| 30 | 40/9 | 10.743 | 0.736 | 0.810 | 9 | 1.000±0.052 | 0.004±0.002 |
| 1 | 32/16 | 10.586 | 0.732 | 0.801 | 15 | 1.000±0.022 | 0.004±0.002 |
| 2 | 29/12 | 10.415 | 0.764 | 0.811 | 10 | 1.000±0.034 | 0.004±0.002 |
| 15 | 49/14 | 10.973 | 0.745 | 0.795 | 14 | 1.000±0.027 | 0.004±0.002 |
| 18 | 20/10 | 10.641 | 0.768 | 0.791 | 9 | 1.000±0.045 | 0.004±0.002 |
| 19 | 36/11 | 10.445 | 0.769 | 0.792 | 10 | 1.000±0.039 | 0.004±0.002 |
| 20 | 19/12 | 10.313 | 0.759 | 0.798 | 12 | 1.000±0.034 | 0.004±0.002 |
| 31 | 40/12 | 10.922 | 0.740 | 0.802 | 12 | 1.000±0.034 | 0.003±0.002 |
| 32 | 40/12 | 10.984 | 0.756 | 0.805 | 12 | 1.000±0.034 | 0.005±0.003 |
| 23 | 47/16 | 10.583 | 0.770 | 0.807 | 15 | 1.000±0.022 | 0.003±0.002 |
| 3 | 26/11 | 10.372 | 0.751 | 0.806 | 10 | 1.000±0.039 | 0.006±0.003 |
| 4 | 34/11 | 10.315 | 0.739 | 0.801 | 11 | 1.000±0.039 | 0.004±0.002 |
| 5 | 56/22 | 10.651 | 0.762 | 0.797 | 22 | 1.000±0.014 | 0.004±0.002 |
| 6 | 53/23 | 10.643 | 0.731 | 0.800 | 23 | 1.000±0.013 | 0.004±0.002 |
| 9 | 26/11 | 10.574 | 0.720 | 0.804 | 10 | 1.000±0.039 | 0.006±0.003 |
| 13 | 38/12 | 10.907 | 0.757 | 0.799 | 12 | 1.000±0.034 | 0.004±0.002 |
| 21 | 47/15 | 11.106 | 0.781 | 0.813 | 14 | 1.000±0.024 | 0.004±0.002 |
| 8 | 30/13 | 10.745 | 0.745 | 0.801 | 13 | 1.000±0.030 | 0.004±0.002 |
| 7 | 27/12 | 10.560 | 0.735 | 0.800 | 10 | 1.000±0.034 | 0.004±0.002 |
| 10 | 40/10 | 10.966 | 0.748 | 0.803 | 10 | 1.000±0.045 | 0.004±0.002 |
| 11 | 33/11 | 10.729 | 0.743 | 0.807 | 11 | 1.000±0.039 | 0.004±0.002 |
| 12 | 24/10 | 10.474 | 0.712 | 0.799 | 10 | 1.000±0.045 | 0.004±0.002 |
| 14 | 30/12 | 10.683 | 0.734 | 0.804 | 11 | 1.000±0.034 | 0.004±0.002 |
| 22 | 40/13 | 10.484 | 0.728 | 0.796 | 12 | 1.000±0.030 | 0.004±0.002 |

n, number of individuals (microsatellites/mitochondrial DNA); AR: allele richness; HO: observed heterozygosity; HE: expected heterozygosity; N: number of haplotypes; h: haplotype diversity; π: nucleotide diversity.
