# Supplementary material for: Population Genetic Studies Revealed Local Adaptation in a High Gene-Flow Marine Fish, the Small Yellow Croaker (Larimichthys polyactis)
Source: PLoS One. 2013 Dec 12;8(12):e83493. doi: 10.1371/journal.pone.0083493 (PMC3861527; doi:10.1371/journal.pone.0083493)
Supplement: Table S5 — Primer sequences used for BAHCC1 gene cloning. (DOCX) [file pone.0083493.s005.docx]

**Table S5** Primer sequences used for BAHCC1 gene cloning.

| Primer | Sequences (from 5' to 3') |
| --- | --- |
| 1F: | CTTCATGTGARTGRGGGAACCAG |
| 1R: | AACTTCTGAGGAWGTYTGAC |
| 2F: | CCTGCCCTRGAGAAATKAATCC |
| 2R: | TTGATTGGCCRCTGCAGCRC |
| 3F: | GTCTTAGTCAGCATTAGCATCA |
| 3R: | GAGCATGCTGATAAGTGGAG |
| 4F: | CTCATCCTGTTACAGTTTC |
| 4R: | GACGGATTGGCAGACAGTTG |
| 5F: | TGTACAGTACCCTCACCTCAAC |
| 5R: | GGTGACAGATGGAAAGGGAATG |
| 6F: | GATCCGCACATTCCCTTTCCATC |
| 6R: | GAGCWGACCTGATTGATYCATCC |
| 7F: | GATTGAAATAACACCTATGA |
| 7R: | GTCGAGCAAAAGAACTCCTT |
| 8F: | GAGCGGTGCTTACAAAGA |
| 8R: | ACAAGGTGCAATGTTRCACG |
| 9F: | CGTGYAACATTGCACCTTG |
| 9R: | TGCTAAATGCTSYRCYATGTTC |
| 10F: | TTGAACATRGYRSAGCATTTAGC |
| 10R: | CTGCTGCGTAGGTGTGTTTG |
| 11F: | AAGTTAGCTCAAACTCCAGT |
| 11R: | TAAAGTGCTTCTCTAGGAACC |
| 12F: | GTATCACCCTCCATCATCGT |
| 12R: | GAATGGAAAAGCCATGCTG |
| 13F: | CCTTATTTCAGCATGGCTTT |
| 13R: | GTGTGTTTTTGTAATGTTTG |
| 14F: | CAAAGACGTGCCTATGTTTTG |
| 14R: | CACAAGGCTAAAACAGCTTC |
| 15F: | GAAGCTGTTTTAGCCTTGTG |
| 15R: | GCAGCAAGGCCAAAGAAATC |
| 16F: | GATTTCTTTGGCCTTGCTGC |
| 16R: | CTACCCAGGGTACAAGGGG |
| 17F: | CAGGGGGTCGTCTTGCACG |
| 17R: | GTTTAGCCTGCAGTAGCAC |
| 18F: | CAAAAACCGTCTGCTGTCTG |
| 18R: | CCATTTGATAAAACCCCACA |
| 19F: | GACAAAGGCTTTCCCACTG |
| 19R: | GCTATTGAAATATATTCAAG |
| 20F: | GACCCAGTGAGAGGCCCTCT |
| 20R: | CGTCTGTTGTGTGTAGGATC |
| 21F: | GTAAAACTAATGAATATGC |
| 21R: | CGGGTAATACCAGAAAATC |
| 22F: | GAGACAGGATACCAAAGCTA |
| 22R: | GAAAGCCGAGATTTCGCTCC |
