# Supplementary material for: Population Genetic Studies Revealed Local Adaptation in a High Gene-Flow Marine Fish, the Small Yellow Croaker (Larimichthys polyactis)
Source: PLoS One. 2013 Dec 12;8(12):e83493. doi: 10.1371/journal.pone.0083493 (PMC3861527; doi:10.1371/journal.pone.0083493)
Supplement: Table S6 — Demographic statistics for mitochondrial DNA. (DOCX) [file pone.0083493.s006.docx]

**Table S6** Demographic statistics for mitochondrial DNA.

|  | Tajima's D | |  | Fu's F_S_ | |  | Mismatch Distribution | | | | |
| --- | --- | --- | --- | --- | --- | --- | --- | --- | --- | --- | --- |
| Group | D | P |  | F_S_ | P |  | SSD | P_SSD_ | Raggedness | P_RAG_ | τ |
| BS | -2.03 | 0.01 |  | -25.385 | 0.00 |  | 0.00068 | 0.872 | 0.01221 | 0.734 | 5.715 |
| NYS | -2.297 | 0.00 |  | -25.354 | 0.00 |  | 0.00989 | 0.011 | 0.01063 | 0.935 | 4.816 |
| CYS | -2.401 | 0.00 |  | -25.176 | 0.00 |  | 0.00082 | 0.326 | 0.01237 | 0.259 | 6.086 |
| SYS | -2.122 | 0.01 |  | -25.041 | 0.00 |  | 0.00157 | 0.887 | 0.00662 | 0.98 | 7.205 |
| NECS | -2.503 | 0.00 |  | -25.077 | 0.00 |  | 0.00222 | 0.059 | 0.00898 | 0.829 | 6.014 |
| CECS | -2.465 | 0.00 |  | -25.227 | 0.00 |  | 0.00045 | 0.784 | 0.00893 | 0.75 | 6.332 |
| TOTAL | -2.446 | 0.00 |  | -24.565 | 0.00 |  | 0.00165 | 0.013 | 0.00894 | 0.954 | 5.807 |

Tajima's D and Fu's FS, corresponding to P value, and SSD and Raggedness corresponding to P value were also indicated; τ: expansion time under mismatch distribution.
