# Supplementary material for: Population Genetic Studies Revealed Local Adaptation in a High Gene-Flow Marine Fish, the Small Yellow Croaker (Larimichthys polyactis)
Source: PLoS One. 2013 Dec 12;8(12):e83493. doi: 10.1371/journal.pone.0083493 (PMC3861527; doi:10.1371/journal.pone.0083493)
Supplement: Table S7 — Sampling location and Genebank accession number for each sample. (DOCX) [file pone.0083493.s007.docx]

**Table S7** Sampling location and Genebank accession number for each sample.

| Individual ID | Sampling ID | COI | CYTB |
| --- | --- | --- | --- |
| 20076816 | 1 | JN250600 | JN243355 |
| 20076817 | 1 | JN250601 | JN243356 |
| 20076818 | 1 | JN250602 | JN243357 |
| 20076819 | 1 | JN250603 | JN243358 |
| 20076830 | 1 | JN250604 | JN243359 |
| 20076831 | 1 | JN250605 | JN243360 |
| 20076832 | 1 | JN250606 | JN243361 |
| 20076833 | 1 | JN250607 | JN243362 |
| 20076834 | 1 | JN250608 | JN243363 |
| 20076837 | 1 | JN250609 | JN243364 |
| 20076838 | 1 | JN250610 | JN243365 |
| 20071657 | 1 | JN250611 | JN243366 |
| 20071658 | 1 | JN250612 | JN243367 |
| 20071659 | 1 | JN250613 | JN243368 |
| 20071660 | 1 | JN250614 | JN243369 |
| 20071661 | 1 | JN250615 | JN243370 |
| 20075601 | 2 | JN250616 | JN243371 |
| 20075602 | 2 | JN250617 | JN243372 |
| 20075603 | 2 | JN250618 | JN243373 |
| 20075604 | 2 | JN250619 | JN243374 |
| 20075605 | 2 | JN250620 | JN243375 |
| 20075606 | 2 | JN250621 | JN243376 |
| 20086566 | 2 | JN250622 | JN243377 |
| 20086567 | 2 | JN250623 | JN243378 |
| 20086568 | 2 | JN250624 | JN243379 |
| 20086569 | 2 | JN250625 | JN243380 |
| 20086570 | 2 | JN250626 | JN243381 |
| 20086571 | 2 | JN250627 | JN243382 |
| 20072919 | 3 | JN250628 | JN243383 |
| 20072920 | 3 | JN250629 | JN243384 |
| 20072921 | 3 | JN250630 | JN243385 |
| 20072922 | 3 | JN250631 | JN243386 |
| 20072923 | 3 | JN250632 | JN243387 |
| 20072924 | 3 | JN250633 | JN243388 |
| 20073275 | 3 | JN250634 | JN243389 |
| 20073276 | 3 | JN250635 | JN243390 |
| 20073277 | 3 | JN250636 | JN243391 |
| 20073278 | 3 | JN250637 | JN243392 |
| 20073279 | 3 | JN250638 | JN243393 |
| 20087971 | 4 | JN250639 | JN243394 |
| 20087972 | 4 | JN250640 | JN243395 |
| 20087973 | 4 | JN250641 | JN243396 |
| 20087975 | 4 | JN250642 | JN243397 |
| 20087977 | 4 | JN250643 | JN243398 |
| 20087978 | 4 | JN250644 | JN243399 |
| 20087979 | 4 | JN250645 | JN243400 |
| 20087980 | 4 | JN250646 | JN243401 |
| 20087982 | 4 | JN250647 | JN243402 |
| 20087983 | 4 | JN250648 | JN243403 |
| 20087984 | 4 | JN250649 | JN243404 |
| 20073771 | 5 | JN250650 | JN243405 |
| 20073772 | 5 | JN250651 | JN243406 |
| 20073773 | 5 | JN250652 | JN243407 |
| 20073774 | 5 | JN250653 | JN243408 |
| 20073775 | 5 | JN250654 | JN243409 |
| 20073776 | 5 | JN250655 | JN243410 |
| 20073777 | 5 | JN250656 | JN243411 |
| 20073778 | 5 | JN250657 | JN243412 |
| 20076611 | 5 | JN250658 | JN243413 |
| 20076612 | 5 | JN250659 | JN243414 |
| 20076613 | 5 | JN250660 | JN243415 |
| 20076614 | 5 | JN250661 | JN243416 |
| 20076615 | 5 | JN250662 | JN243417 |
| 20076616 | 5 | JN250663 | JN243418 |
| 20076618 | 5 | JN250664 | JN243419 |
| 20087811 | 5 | JN250665 | JN243420 |
| 20087812 | 5 | JN250666 | JN243421 |
| 20087813 | 5 | JN250667 | JN243422 |
| 20087814 | 5 | JN250668 | JN243423 |
| 20087815 | 5 | JN250669 | JN243424 |
| 20087816 | 5 | JN250670 | JN243425 |
| 20087818 | 5 | JN250671 | JN243426 |
| 20089161 | 6 | JN250672 | JN243427 |
| 20089162 | 6 | JN250673 | JN243428 |
| 20089163 | 6 | JN250674 | JN243429 |
| 20089164 | 6 | JN250675 | JN243430 |
| 20089165 | 6 | JN250676 | JN243431 |
| 20089166 | 6 | JN250677 | JN243432 |
| 20089167 | 6 | JN250678 | JN243433 |
| 20089168 | 6 | JN250679 | JN243434 |
| 20089169 | 6 | JN250680 | JN243435 |
| 20089170 | 6 | JN250681 | JN243436 |
| 20089171 | 6 | JN250682 | JN243437 |
| 20089172 | 6 | JN250683 | JN243438 |
| 20089221 | 6 | JN250684 | JN243439 |
| 20089222 | 6 | JN250685 | JN243440 |
| 20089223 | 6 | JN250686 | JN243441 |
| 20089224 | 6 | JN250687 | JN243442 |
| 20089225 | 6 | JN250688 | JN243443 |
| 20089226 | 6 | JN250689 | JN243444 |
| 20089227 | 6 | JN250690 | JN243445 |
| 20089228 | 6 | JN250691 | JN243446 |
| 20089229 | 6 | JN250692 | JN243447 |
| 20089231 | 6 | JN250693 | JN243448 |
| 20089232 | 6 | JN250694 | JN243449 |
| 20087911 | 7 | JN250695 | JN243450 |
| 20087912 | 7 | JN250696 | JN243451 |
| 20087913 | 7 | JN250697 | JN243452 |
| 20087915 | 7 | JN250698 | JN243453 |
| 20087916 | 7 | JN250699 | JN243454 |
| 20087917 | 7 | JN250700 | JN243455 |
| 20087919 | 7 | JN250701 | JN243456 |
| 20087920 | 7 | JN250702 | JN243457 |
| 20087930 | 7 | JN250703 | JN243458 |
| 20087931 | 7 | JN250704 | JN243459 |
| 20087932 | 7 | JN250705 | JN243460 |
| 20087933 | 7 | JN250706 | JN243461 |
| 200811251 | 8 | JN250707 | JN243462 |
| 200811252 | 8 | JN250708 | JN243463 |
| 200811253 | 8 | JN250709 | JN243464 |
| 200811254 | 8 | JN250710 | JN243465 |
| 200811255 | 8 | JN250711 | JN243466 |
| 200811256 | 8 | JN250712 | JN243467 |
| 200811257 | 8 | JN250713 | JN243468 |
| 200811258 | 8 | JN250714 | JN243469 |
| 200811271 | 8 | JN250715 | JN243470 |
| 200811272 | 8 | JN250716 | JN243471 |
| 200811273 | 8 | JN250717 | JN243472 |
| 200811274 | 8 | JN250718 | JN243473 |
| 200811275 | 8 | JN250719 | JN243474 |
| 200811039 | 9 | JN250720 | JN243475 |
| 200811040 | 9 | JN250721 | JN243476 |
| 200811041 | 9 | JN250722 | JN243477 |
| 200811045 | 9 | JN250723 | JN243478 |
| 200811046 | 9 | JN250724 | JN243479 |
| 200811047 | 9 | JN250725 | JN243480 |
| 200811054 | 9 | JN250726 | JN243481 |
| 200811055 | 9 | JN250727 | JN243482 |
| 200811061 | 9 | JN250728 | JN243483 |
| 200811062 | 9 | JN250729 | JN243484 |
| 200811063 | 9 | JN250730 | JN243485 |
| 200811092 | 10 | JN250731 | JN243486 |
| 200811093 | 10 | JN250732 | JN243487 |
| 200811094 | 10 | JN250733 | JN243488 |
| 200811096 | 10 | JN250734 | JN243489 |
| 200811111 | 10 | JN250735 | JN243490 |
| 200811113 | 10 | JN250736 | JN243491 |
| 200811115 | 10 | JN250737 | JN243492 |
| 200811116 | 10 | JN250738 | JN243493 |
| 200811117 | 10 | JN250739 | JN243494 |
| 200811118 | 10 | JN250740 | JN243495 |
| 200811132 | 11 | JN250741 | JN243496 |
| 200811133 | 11 | JN250742 | JN243497 |
| 200811134 | 11 | JN250743 | JN243498 |
| 200811137 | 11 | JN250744 | JN243499 |
| 200811148 | 11 | JN250745 | JN243500 |
| 200811149 | 11 | JN250746 | JN243501 |
| 200811150 | 11 | JN250747 | JN243502 |
| 200811151 | 11 | JN250748 | JN243503 |
| 200811161 | 11 | JN250749 | JN243504 |
| 200811162 | 11 | JN250750 | JN243505 |
| 200811163 | 11 | JN250751 | JN243506 |
| 200811071 | 12 | JN250752 | JN243507 |
| 200811072 | 12 | JN250753 | JN243508 |
| 200811073 | 12 | JN250754 | JN243509 |
| 200811074 | 12 | JN250755 | JN243510 |
| 200811075 | 12 | JN250756 | JN243511 |
| 200811076 | 12 | JN250757 | JN243512 |
| 200811081 | 12 | JN250758 | JN243513 |
| 200811082 | 12 | JN250759 | JN243514 |
| 200811083 | 12 | JN250760 | JN243515 |
| 200811084 | 12 | JN250761 | JN243516 |
| 200812491 | 13 | JN250762 | JN243517 |
| 200812492 | 13 | JN250763 | JN243518 |
| 200812493 | 13 | JN250764 | JN243519 |
| 200812495 | 13 | JN250765 | JN243520 |
| 200812496 | 13 | JN250766 | JN243521 |
| 200812497 | 13 | JN250767 | JN243522 |
| 200812507 | 13 | JN250768 | JN243523 |
| 200812508 | 13 | JN250769 | JN243524 |
| 200812509 | 13 | JN250770 | JN243525 |
| 200812510 | 13 | JN250771 | JN243526 |
| 200812511 | 13 | JN250772 | JN243527 |
| 200812512 | 13 | JN250773 | JN243528 |
| 200810971 | 14 | JN250774 | JN243529 |
| 200810972 | 14 | JN250775 | JN243530 |
| 200810973 | 14 | JN250776 | JN243531 |
| 200810974 | 14 | JN250777 | JN243532 |
| 200810975 | 14 | JN250778 | JN243533 |
| 200810976 | 14 | JN250779 | JN243534 |
| 200810977 | 14 | JN250780 | JN243535 |
| 200810978 | 14 | JN250781 | JN243536 |
| 200810986 | 14 | JN250782 | JN243537 |
| 200810987 | 14 | JN250783 | JN243538 |
| 200810989 | 14 | JN250784 | JN243539 |
| 200810991 | 14 | JN250785 | JN243540 |
| 200905381 | 15 | JN250786 | JN243541 |
| 200905383 | 15 | JN250787 | JN243542 |
| 200905384 | 15 | JN250788 | JN243543 |
| 200905385 | 15 | JN250789 | JN243544 |
| 200905386 | 15 | JN250790 | JN243545 |
| 200905387 | 15 | JN250791 | JN243546 |
| 200905401 | 15 | JN250792 | JN243547 |
| 200905403 | 15 | JN250793 | JN243548 |
| 200905404 | 15 | JN250794 | JN243549 |
| 200905405 | 15 | JN250795 | JN243550 |
| 200905406 | 15 | JN250796 | JN243551 |
| 200905471 | 15 | JN250797 | JN243552 |
| 200905472 | 15 | JN250798 | JN243553 |
| 200905476 | 15 | JN250799 | JN243554 |
| 200905481 | 16 | JN250800 | JN243555 |
| 200905482 | 16 | JN250801 | JN243556 |
| 200905483 | 16 | JN250802 | JN243557 |
| 200905484 | 16 | JN250803 | JN243558 |
| 200905485 | 16 | JN250804 | JN243559 |
| 200905486 | 16 | JN250805 | JN243560 |
| 200905493 | 16 | JN250806 | JN243561 |
| 200905494 | 16 | JN250807 | JN243562 |
| 200905495 | 16 | JN250808 | JN243563 |
| 200905496 | 16 | JN250809 | JN243564 |
| 200905881 | 17 | JN250810 | JN243565 |
| 200905884 | 17 | JN250811 | JN243566 |
| 200905885 | 17 | JN250812 | JN243567 |
| 200905886 | 17 | JN250813 | JN243568 |
| 200905891 | 17 | JN250814 | JN243569 |
| 200905892 | 17 | JN250815 | JN243570 |
| 200905893 | 17 | JN250816 | JN243571 |
| 200905894 | 17 | JN250817 | JN243572 |
| 200905895 | 17 | JN250818 | JN243573 |
| 200905906 | 17 | JN250819 | JN243574 |
| 200905907 | 17 | JN250820 | JN243575 |
| 200905908 | 17 | JN250821 | JN243576 |
| 200907661 | 18 | JN250822 | JN243577 |
| 200907662 | 18 | JN250823 | JN243578 |
| 200907663 | 18 | JN250824 | JN243579 |
| 200907664 | 18 | JN250825 | JN243580 |
| 200907665 | 18 | JN250826 | JN243581 |
| 200907666 | 18 | JN250827 | JN243582 |
| 200907671 | 18 | JN250828 | JN243583 |
| 200907672 | 18 | JN250829 | JN243584 |
| 200907674 | 18 | JN250830 | JN243585 |
| 200907676 | 18 | JN250831 | JN243586 |
| 200908801 | 19 | JN250832 | JN243587 |
| 200908802 | 19 | JN250833 | JN243588 |
| 200908803 | 19 | JN250834 | JN243589 |
| 200908804 | 19 | JN250835 | JN243590 |
| 200908806 | 19 | JN250836 | JN243591 |
| 200908807 | 19 | JN250837 | JN243592 |
| 200908808 | 19 | JN250838 | JN243593 |
| 200908823 | 19 | JN250839 | JN243594 |
| 200908824 | 19 | JN250840 | JN243595 |
| 200908825 | 19 | JN250841 | JN243596 |
| 200908826 | 19 | JN250842 | JN243597 |
| 200907316 | 20 | JN250843 | JN243598 |
| 200907317 | 20 | JN250844 | JN243599 |
| 200907318 | 20 | JN250845 | JN243600 |
| 200907319 | 20 | JN250846 | JN243601 |
| 200907320 | 20 | JN250847 | JN243602 |
| 200907321 | 20 | JN250848 | JN243603 |
| 200907326 | 20 | JN250849 | JN243604 |
| 200907327 | 20 | JN250850 | JN243605 |
| 200907328 | 20 | JN250851 | JN243606 |
| 200907329 | 20 | JN250852 | JN243607 |
| 200907330 | 20 | JN250853 | JN243608 |
| 200907331 | 20 | JN250854 | JN243609 |
| X11 | 21 | JN250855 | JN243610 |
| X12 | 21 | JN250856 | JN243611 |
| X13 | 21 | JN250857 | JN243612 |
| X14 | 21 | JN250858 | JN243613 |
| X15 | 21 | JN250859 | JN243614 |
| X16 | 21 | JN250860 | JN243615 |
| X18 | 21 | JN250861 | JN243616 |
| X81 | 21 | JN250862 | JN243617 |
| X82 | 21 | JN250863 | JN243618 |
| X83 | 21 | JN250864 | JN243619 |
| X84 | 21 | JN250865 | JN243620 |
| X85 | 21 | JN250866 | JN243621 |
| X86 | 21 | JN250867 | JN243622 |
| X87 | 21 | JN250868 | JN243623 |
| X88 | 21 | JN250869 | JN243624 |
| 201004032 | 22 | JN250870 | JN243625 |
| 201004033 | 22 | JN250871 | JN243626 |
| 201004034 | 22 | JN250872 | JN243627 |
| 201004035 | 22 | JN250873 | JN243628 |
| 201004036 | 22 | JN250874 | JN243629 |
| 201004051 | 22 | JN250875 | JN243630 |
| 201004052 | 22 | JN250876 | JN243631 |
| 201004053 | 22 | JN250877 | JN243632 |
| 201004055 | 22 | JN250878 | JN243633 |
| 201004065 | 22 | JN250879 | JN243634 |
| 201004067 | 22 | JN250880 | JN243635 |
| 201004068 | 22 | JN250881 | JN243636 |
| 201004069 | 22 | JN250882 | JN243637 |
| 201004471 | 23 | JN250883 | JN243638 |
| 201004472 | 23 | JN250884 | JN243639 |
| 201004473 | 23 | JN250885 | JN243640 |
| 201004474 | 23 | JN250886 | JN243641 |
| 201004475 | 23 | JN250887 | JN243642 |
| 201004476 | 23 | JN250888 | JN243643 |
| 201004477 | 23 | JN250889 | JN243644 |
| 201004478 | 23 | JN250890 | JN243645 |
| 201004491 | 23 | JN250891 | JN243646 |
| 201004492 | 23 | JN250892 | JN243647 |
| 201004493 | 23 | JN250893 | JN243648 |
| 201004494 | 23 | JN250894 | JN243649 |
| 201004495 | 23 | JN250895 | JN243650 |
| 201004496 | 23 | JN250896 | JN243651 |
| 201004497 | 23 | JN250897 | JN243652 |
| 201004498 | 23 | JN250898 | JN243653 |
| 20074261 | 24 | JN250899 | JN243654 |
| 20074262 | 24 | JN250900 | JN243655 |
| 20074263 | 24 | JN250901 | JN243656 |
| 20074264 | 24 | JN250902 | JN243657 |
| 20074265 | 24 | JN250903 | JN243658 |
| 319401 | 25 | JN250904 | JN243659 |
| 319402 | 25 | JN250905 | JN243660 |
| 319404 | 25 | JN250906 | JN243661 |
| 319405 | 25 | JN250907 | JN243662 |
| 319408 | 25 | JN250908 | JN243663 |
| 319409 | 25 | JN250909 | JN243664 |
| 319410 | 25 | JN250910 | JN243665 |
| 319413 | 25 | JN250911 | JN243666 |
| 319415 | 25 | JN250912 | JN243667 |
| 355101 | 26 | JN250913 | JN243668 |
| 355105 | 26 | JN250914 | JN243669 |
| 355106 | 26 | JN250915 | JN243670 |
| 355107 | 26 | JN250916 | JN243671 |
| 355108 | 26 | JN250917 | JN243672 |
| 355109 | 26 | JN250918 | JN243673 |
| 355111 | 26 | JN250919 | JN243674 |
| 358401 | 26 | JN250920 | JN243675 |
| 358403 | 26 | JN250921 | JN243676 |
| 358404 | 26 | JN250922 | JN243677 |
| 358405 | 26 | JN250923 | JN243678 |
| 358406 | 26 | JN250924 | JN243679 |
| 358407 | 26 | JN250925 | JN243680 |
| 449402 | 27 | JN250926 | JN243681 |
| 449403 | 27 | JN250927 | JN243682 |
| 449404 | 27 | JN250928 | JN243683 |
| 449408 | 27 | JN250929 | JN243684 |
| 449409 | 27 | JN250930 | JN243685 |
| 449410 | 27 | JN250931 | JN243686 |
| 449411 | 27 | JN250932 | JN243687 |
| 449412 | 27 | JN250933 | JN243688 |
| 449414 | 27 | JN250934 | JN243689 |
| 449416 | 27 | JN250935 | JN243690 |
| 589404 | 28 | JN250936 | JN243691 |
| 589405 | 28 | JN250937 | JN243692 |
| 589406 | 28 | JN250938 | JN243693 |
| 589407 | 28 | JN250939 | JN243694 |
| 589408 | 28 | JN250940 | JN243695 |
| 589409 | 28 | JN250941 | JN243696 |
| 589410 | 28 | JN250942 | JN243697 |
| 589411 | 28 | JN250943 | JN243698 |
| 589413 | 28 | JN250944 | JN243699 |
| 589414 | 28 | JN250945 | JN243700 |
| 589415 | 28 | JN250946 | JN243701 |
| 589416 | 28 | JN250947 | JN243702 |
| 625101 | 29 | JN250948 | JN243703 |
| 625102 | 29 | JN250949 | JN243704 |
| 625103 | 29 | JN250950 | JN243705 |
| 625104 | 29 | JN250951 | JN243706 |
| 625106 | 29 | JN250952 | JN243707 |
| 625107 | 29 | JN250953 | JN243708 |
| 625109 | 29 | JN250954 | JN243709 |
| 625110 | 29 | JN250955 | JN243710 |
| 625111 | 29 | JN250956 | JN243711 |
| 625112 | 29 | JN250957 | JN243712 |
| 625113 | 29 | JN250958 | JN243713 |
| 625114 | 29 | JN250959 | JN243714 |
| 625115 | 29 | JN250960 | JN243715 |
| 625116 | 29 | JN250961 | JN243716 |
| 665104 | 30 | JN250962 | JN243717 |
| 665105 | 30 | JN250963 | JN243718 |
| 665106 | 30 | JN250964 | JN243719 |
| 665108 | 30 | JN250965 | JN243720 |
| 665112 | 30 | JN250966 | JN243721 |
| 665113 | 30 | JN250967 | JN243722 |
| 665114 | 30 | JN250968 | JN243723 |
| 665115 | 30 | JN250969 | JN243724 |
| 665116 | 30 | JN250970 | JN243725 |
| 749401 | 31 | JN250971 | JN243726 |
| 749402 | 31 | JN250972 | JN243727 |
| 749403 | 31 | JN250973 | JN243728 |
| 749404 | 31 | JN250974 | JN243729 |
| 749405 | 31 | JN250975 | JN243730 |
| 749406 | 31 | JN250976 | JN243731 |
| 749407 | 31 | JN250977 | JN243732 |
| 749408 | 31 | JN250978 | JN243733 |
| 749411 | 31 | JN250979 | JN243734 |
| 749412 | 31 | JN250980 | JN243735 |
| 749414 | 31 | JN250981 | JN243736 |
| 749416 | 31 | JN250982 | JN243737 |
| RZ01 | 32 | JN250983 | JN243738 |
| RZ03 | 32 | JN250984 | JN243739 |
| RZ04 | 32 | JN250985 | JN243740 |
| RZ05 | 32 | JN250986 | JN243741 |
| RZ06 | 32 | JN250987 | JN243742 |
| RZ07 | 32 | JN250988 | JN243743 |
| RZ09 | 32 | JN250989 | JN243744 |
| RZ10 | 32 | JN250990 | JN243745 |
| RZ11 | 32 | JN250991 | JN243746 |
| RZ12 | 32 | JN250992 | JN243747 |
| RZ13 | 32 | JN250993 | JN243748 |
| RZ16 | 32 | JN250994 | JN243749 |
