# Supplementary figures and images for: Population Genetic Studies Revealed Local Adaptation in a High Gene-Flow Marine Fish, the Small Yellow Croaker (Larimichthys polyactis)
Source: PLoS One. 2013 Dec 12;8(12):e83493. doi: 10.1371/journal.pone.0083493 (PMC3861527; doi:10.1371/journal.pone.0083493)

**Figure S1** ΔK values calculated according to Evanno *et al.* (2005) for 20 and 22 loci data sets, respectively.


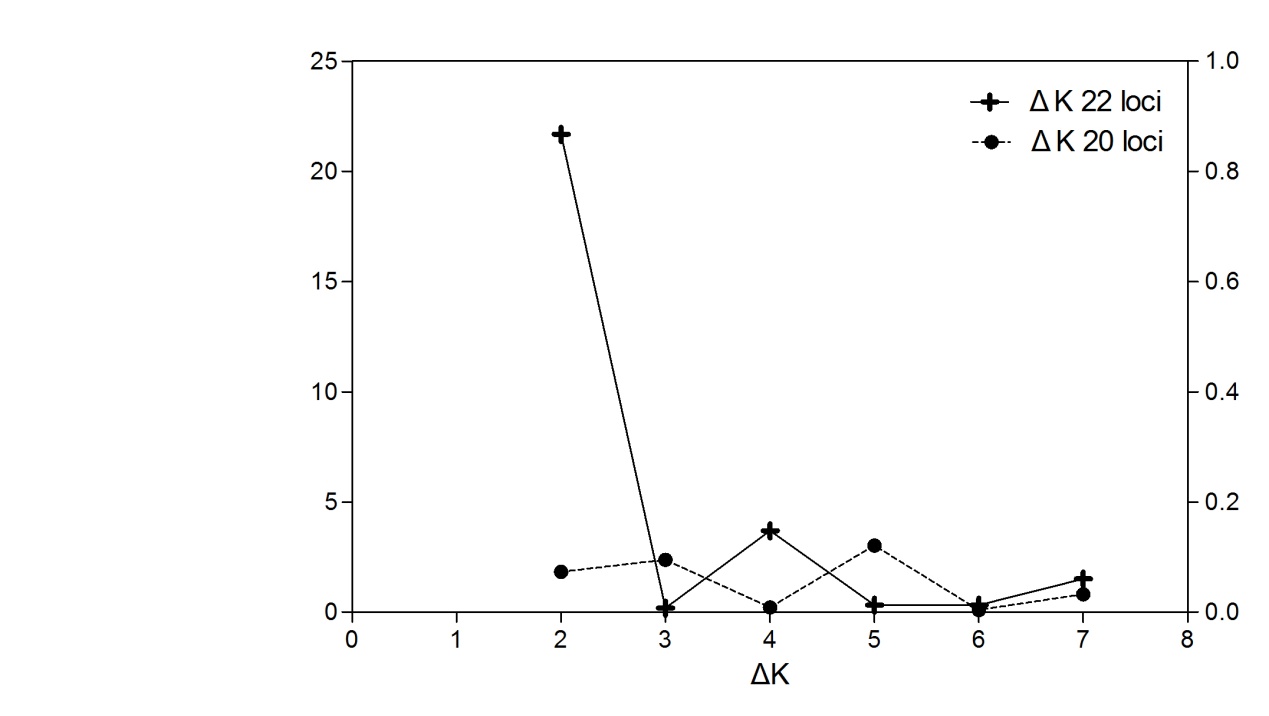

Supplement: Figure S1 — ΔK values calculated according to Evanno et al. (2005) for 20 and 22 loci data sets, respectively. (DOCX) [file pone.0083493.s008.docx]

**Figure S2** Sequence alignment of BAHCC1 protein sequences among several species.


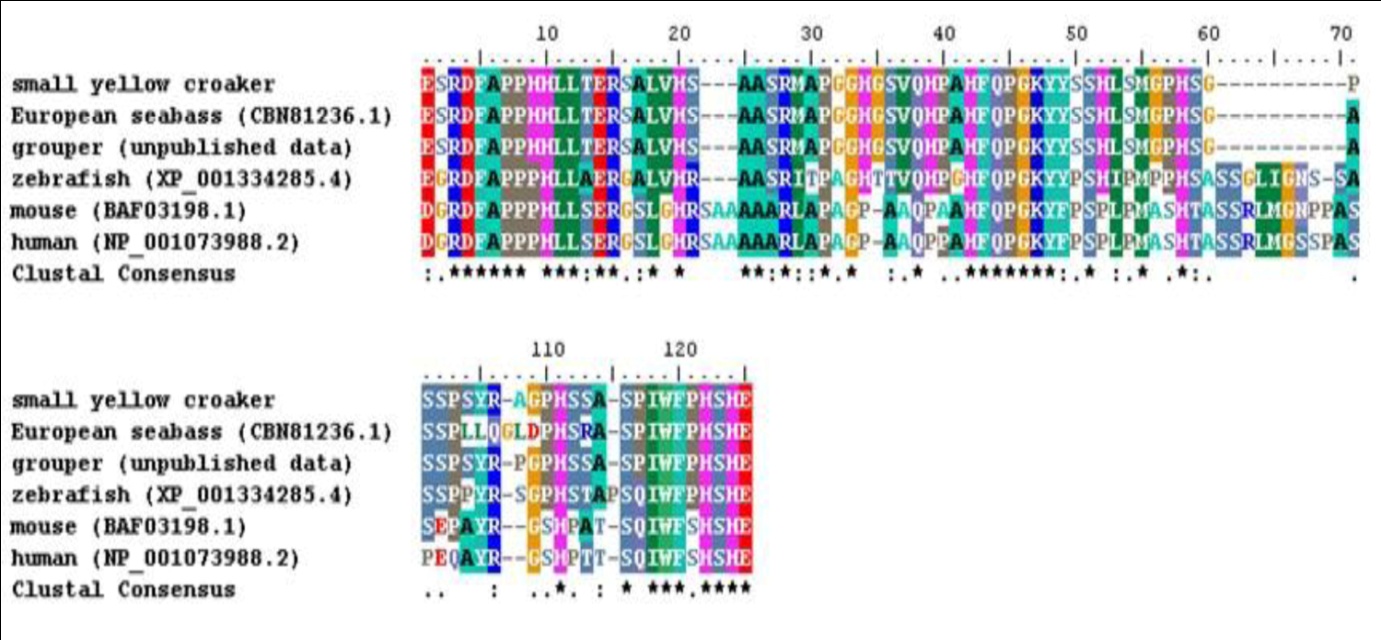

Supplement: Figure S2 — Sequence alignment of BAHCC1 protein sequences among several species. (DOCX) [file pone.0083493.s009.docx]
