# Supplementary material for: Population Genetic Studies Revealed Local Adaptation in a High Gene-Flow Marine Fish, the Small Yellow Croaker (Larimichthys polyactis)
Source: PLoS One. 2013 Dec 12;8(12):e83493. doi: 10.1371/journal.pone.0083493 (PMC3861527; doi:10.1371/journal.pone.0083493)

**Figure S3** Phylogenetic tree of the small yellow croaker (*Larimichthys polyactis*) based on mitochondrial DNA haplotypes. Bootstrap supports of more than 50% from 1000 replicates are shown. The Large yellow croaker (*Larimichthys crocea*) is used as outgroup (GenBank no. EU339149).

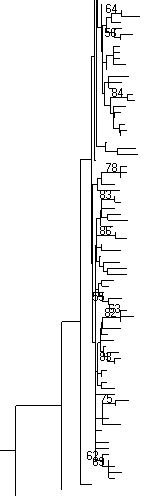

Supplement: Figure S3 — Phylogenetic tree of the small yellow croaker (Larimichthys polyactis) based on mitochondrial DNA haplotypes. Bootstrap supports of more than 50% from 1000 replicates are shown. The Large yellow croaker (Larimichthys crocea) is used as outgroup (GenBank no. EU339149). (DOCX) [file pone.0083493.s010.docx]
